# Mondo complexes regulate TFEB via TOR inhibition to promote longevity in response to gonadal signals

**DOI:** 10.1038/ncomms10944

**Published:** 2016-03-22

**Authors:** Shuhei Nakamura, Özlem Karalay, Philipp S. Jäger, Makoto Horikawa, Corinna Klein, Kayo Nakamura, Christian Latza, Sven E. Templer, Christoph Dieterich, Adam Antebi

**Affiliations:** 1 Department of Molecular Genetics of Ageing, Max Planck Institute for Biology of Ageing, Joseph Stelzmann Strasse 9b, Cologne 50931, Germany; 2 Computational RNA Biology and Ageing, Max Planck Institute for Biology of Ageing, Cologne 50931, Germany; 3 Department of Molecular and Cellular Biology, Huffington Center on Aging, Baylor College of Medicine, Houston, Texas 77030, USA; 4 Cologne Excellence Cluster on Cellular Stress Responses in Aging-Associated Diseases (CECAD), University of Cologne, 50674 Cologne, Germany

## Abstract

Germline removal provokes longevity in several species and shifts resources towards survival and repair. Several *Caenorhabditis elegans* transcription factors regulate longevity arising from germline removal; yet, how they work together is unknown. Here we identify a Myc-like HLH transcription factor network comprised of Mondo/Max-like complex (MML-1/MXL-2) to be required for longevity induced by germline removal, as well as by reduced TOR, insulin/IGF signalling and mitochondrial function. Germline removal increases MML-1 nuclear accumulation and activity. Surprisingly, MML-1 regulates nuclear localization and activity of HLH-30/TFEB, a convergent regulator of autophagy, lysosome biogenesis and longevity, by downregulating TOR signalling via LARS-1/leucyl-transfer RNA synthase. HLH-30 also upregulates MML-1 upon germline removal. Mammalian MondoA/B and TFEB show similar mutual regulation. MML-1/MXL-2 and HLH-30 transcriptomes show both shared and preferential outputs including MDL-1/MAD-like HLH factor required for longevity. These studies reveal how an extensive interdependent HLH transcription factor network distributes responsibility and mutually enforces states geared towards reproduction or survival.

## Abstract (web summary)

Removal of the *C. elegans* germline substantially increases organismal lifespan. Here, Nakamura *et al*. show that the transcription factors MML-1 and MXL-2 coordinate this process in that they reduce TOR signalling and increase autophagy by regulating activity of HLH-30.

## Graphical abstract

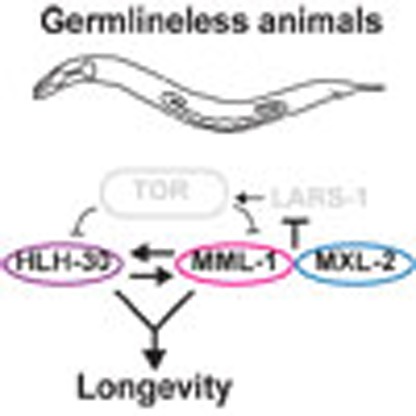

Pioneering work in model genetic organisms has led to the discovery of conserved signalling pathways that regulate animal longevity^1^. These pathways include reduced insulin/insulin growth factor (IGF) signalling, dietary restriction, reduced mitochondrial respiration, decreased target of rapamycin (TOR) signalling and signals from the reproductive system, which promote health and longevity through enhanced protein quality control, stress resistance, metabolic homeostasis and immunity. Molecular genetic experiments performed in *C. elegans* reveal that these pathways work through specific constellations of transcription factors. For example, the long life of reduced insulin/IGF signalling *daf-2*/InsulinR mutants depends largely on the DAF-16/FOXO, SKN-1/NFE-2 and HSF-1 transcription factors^2^,^3^,^4^, whereas dietary restriction-induced longevity is mediated by PHA-4/FOXA, NHR-62/HNF4, SKN-1/NFE-2 and NHR-8/LXR^5^,^6^,^7^,^8^.

Of particular interest is the gonadal longevity pathway. Removal of germline precursors results in a prodigious 60% increase in lifespan^9^, which depends on bile acid-like steroids emanating from the somatic gonad^10^,^11^,^12^. Virtually, every known longevity factor including the steroid receptor DAF-12/FXR, DAF-16/FOXO, HSF-1, NHR-80/HNF4, PHA-4/FOXA and NHR-49/PPARα function in this pathway^4^,^9^,^13^,^14^,^15^ but how these factors cooperate in transcriptional networks is poorly understood. Dissecting such circuitry may reveal how regulatory functions are distributed and converge at key nodes within networks to establish signalling states and help pinpoint processes critical for longevity.

Although the major longevity pathways behave independently by genetic criteria, a number of convergent processes have emerged in common. This includes the process of autophagy, the intracellular engulfment of protein aggregates, defective organelles and internal membranes, with delivery to the lysosome for turnover. Autophagy is necessary for longevity in many pathways and, in some cases, enhanced autophagy is sufficient to extend life^16^,^17^,^18^,^19^,^20^. HLH-30/TFEB transcription factor, recently shown to promote autophagy and lysosomal biogenesis^21^,^22^, also works in various longevity pathways, revealing a key regulator of core longevity mechanisms^19^. However, the regulatory cascades that govern these core longevity mechanisms remain unexplored.

To uncover new regulators of the gonadal longevity pathway, we performed systematic RNA interference (RNAi) screens and discovered a key role for Myc superfamily members MML-1 (Myc/Mondo-like) and its partner MXL-2 (Max, Max-like) in mediating gonadal longevity, as well as longevity in other conserved pathways. Our studies suggest that MML-1 and MXL-2 work in transcriptional cascades that reduce TOR activity, which in turn stimulates autophagy and HLH-30/TFEB activity in the nucleus. Together, MML-1/MXL-2 and HLH-30 cooperate to extend life but also regulate distinct gene sets important for longevity. These studies illuminate a core regulatory network broadly affecting animal lifespan.

## Results

### *mml-1/mxl-2* are required for multiple longevity pathways

**Figure 1:**
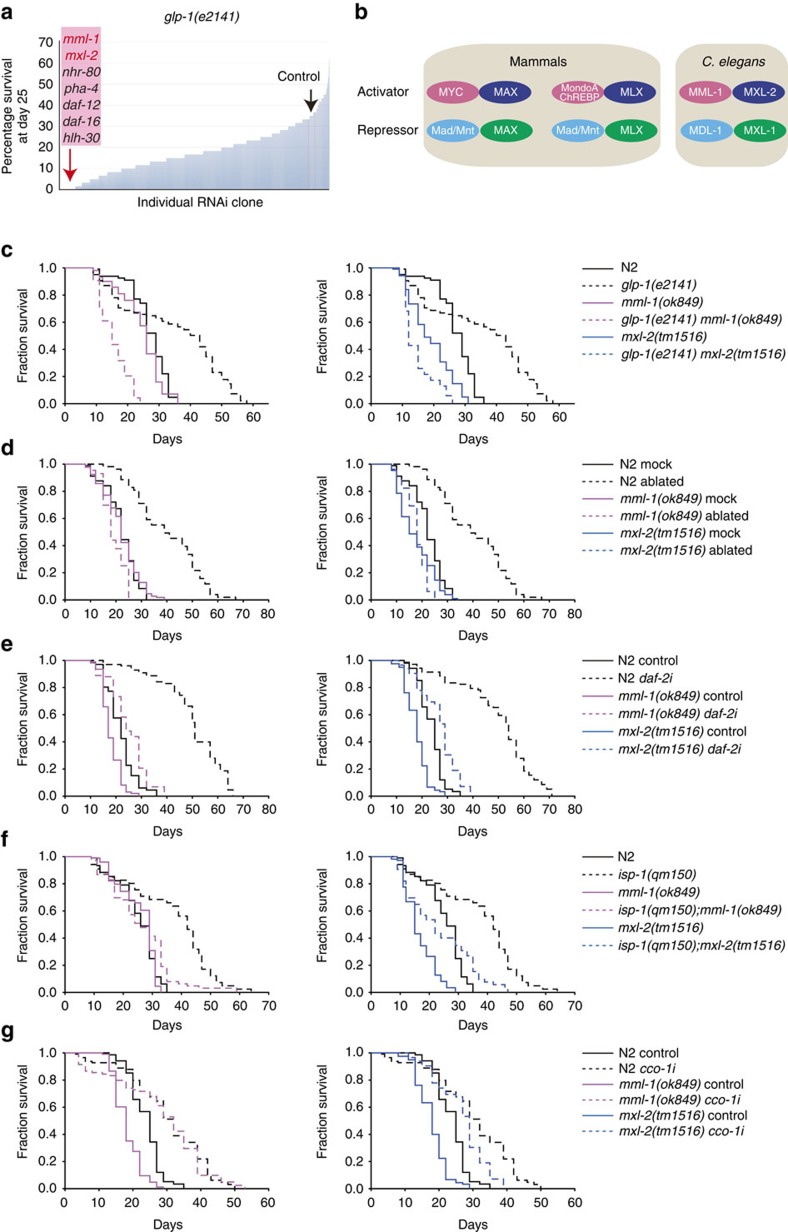
MML-1/MXL-2 are required for multiple longevity pathways. (**a**) *glp-1(e2141)* suppressor screens identify *mml-1* and *mxl-2* as new transcription factors required for gonadal longevity. Survivorship of *glp-1(e2141)* worms on control RNAi (L4440, black arrow) and experimental RNAi was determined at day 25. Knockdown of known factors (for example, *daf-16*, *daf-12* and *hlh-30*), as well as *mml-1* and *mxl-2*, completely suppressed *glp-1* survivorship. Survivorship of 60 worms was determined and screening conducted twice. (**b**) *C. elegans* Myc superfamily comprises MML-1/MXL-2 and MDL-1/MXL-1 complexes. (**c–g**) Roles of *mml-1* and *mxl-2* in various longevity pathways. *mml-1(ok849)* and *mxl-2(tm1516)* deletions suppress longevity of germline-deficient *glp-1(e2141)* mutants (**c**) and animals with germline laser microsurgery (**d**). *mml-1* and *mxl-2* suppress longevity of *daf-2* RNAi (**e**). *mml-1* is required for *isp-1 (qm150)* longevity **(f)** but not required for the longevity conferred by *cco-1* RNAi (**g**). *mxl-2* is not specifically required for *isp-1* or *cco-1* RNAi longevity (**f**,**g**). See Supplementary Table 1 for details and repeats.

To identify new mediators of gonadal longevity, we performed RNAi screens encompassing *ca*. 600 transcription factors for suppressors of longevity in germline-less *glp-1* mutants (Fig. 1a). We obtained previously known regulators such as *daf-16*, *daf-12* and *hlh-30*, as well as several novel regulators including *mml-1* and *mxl-2* as potent candidates. MML-1 and MXL-2 belong to the Myc super family of basic helix-loop-helix (bHLH-Zip) E-box factors: MML-1 (Myc and Mondo-like) is homologous to MondoA/ChREBP, whereas MXL-2 is homologous to Max-like and works together with MML-1 in an activation complex^23^(Fig. 1b). In mammals, MondoA/Max-like and ChREBP (MondoB)/Max-like complexes respond to glucose and regulate glycolysis and lipogenesis^24^,^25^.

Demographic analysis confirmed that *mml-1* and *mxl-2* deletions abolished lifespan extension in *glp-1* mutants and in animals whose germline precursors were removed by laser microsurgery, while only modestly shortening wild-type lifespan (Fig. 1c,d). We also examined interactions with other longevity pathways and found that *mml-1* and *mxl-2* were largely required for *daf-2*/InsR longevity (Fig. 1e), similar to previous reports^26^. In addition, *mml-1* was partially required for *isp-1* longevity but had little effect on longevity on *cco-1* RNAi, whereas *mxl-2* had little specific effect on either (Fig. 1f,g). Consistent with a role in longevity regulation, *mml-1* overexpression in wild type sufficed to extend lifespan in three of six experiments (Supplementary Fig. 1a and Supplementary Table 1) but did not further extend *glp-1* longevity (Supplementary Fig. 1b). These findings indicate that *mml-1* and *mxl-2* work within several longevity pathways and have overlapping but distinct functions.

### Signals from the reproductive system regulate *mml-1*

**Figure 2:**
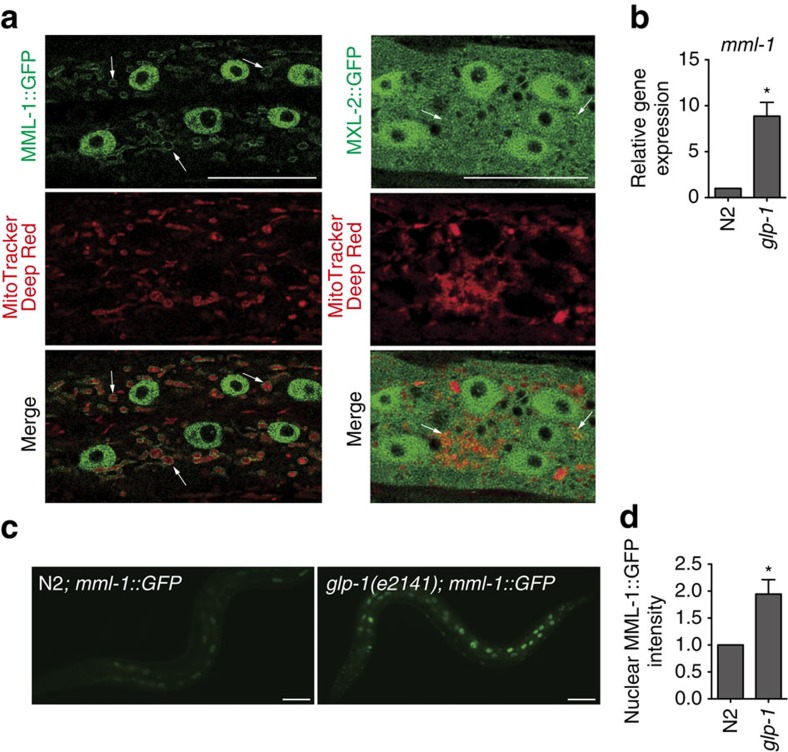
MML-1 is regulated on germline removal. (**a**) MML-1::GFP and MXL-2::GFP (green) in hypodermal cells (wild type, L4 stage). MML-1::GFP is localized to the nucleus and the mitochondria (white arrows). MXL-2::GFP is not specifically localized, but occasionally overlapped with the mitochondria (white arrows). Mitochondria are marked by MitoTracker Deep Red (Red). (**b**) *mml-1* transcripts are upregulated in *glp-1(e2141)* animals relative to wild-type N2 as measured by qRT–PCR using total RNA samples prepared from >200 young adult worms. Mean±s.e.m. from three independent experiments are depicted and are normalized to wild-type N2. *P*-value (*P<0.05) was determined by *t*-test. (**c**) The representative fluorescent images of *mml-1*::GFP transgenic worms in wild-type N2 and *glp-1* worms at day 1 young adult stage. Nuclear MML-1::GFP is elevated in *glp-1* animals. (**d**) Quantification of nuclear MML-1::GFP in the posterior intestinal nucleus at day 1 young adult stage. Mean±s.e.m. from four biological replicates (>20 worms each) relative to N2 are presented. *P*-value (*P<0.05) was determined by *t*-test. Two other independent MML-1::GFP transgenic lines show a similar tendency (not shown). Scale bars, 20 μm (**a**) and 50 μm (**c**).

A rescuing MML-1::GFP translational reporter (Supplementary Fig. 1c) was widely expressed and found in the cytoplasm and nuclei of the intestine, neurons, muscle, hypodermis, excretory cell and other tissues (Supplementary Fig. 2a)^23^. A closer examination of the subcellular localization revealed that MML-1::GFP also co-localized with the mitochondria, comparable to mammalian MondoA (Fig. 2a)^27^. A rescuing MXL-2::GFP translational reporter construct was also widely expressed and overlapped with MML-1 in most tissues (Fig.2a and Supplementary Figs 1d and 2b), but was distributed smoothly in the cytoplasm and nucleus, with only sporadic mitochondrial localization. Interestingly, we noticed that both *mml-1* messenger RNA levels and MML-1::GFP nuclear accumulation were increased in *glp-1* mutants (Fig. 2b-d). In contrast, MXL-2::GFP did not change on germline removal, although *mxl-2* transcripts were increased *ca*. 1.5-fold (Supplementary Fig. 2c–e). Thus, *mml-1* is visibly regulated in response to germline signalling.

### Mutual regulation of MML-1 and HLH-30

**Figure 3:**
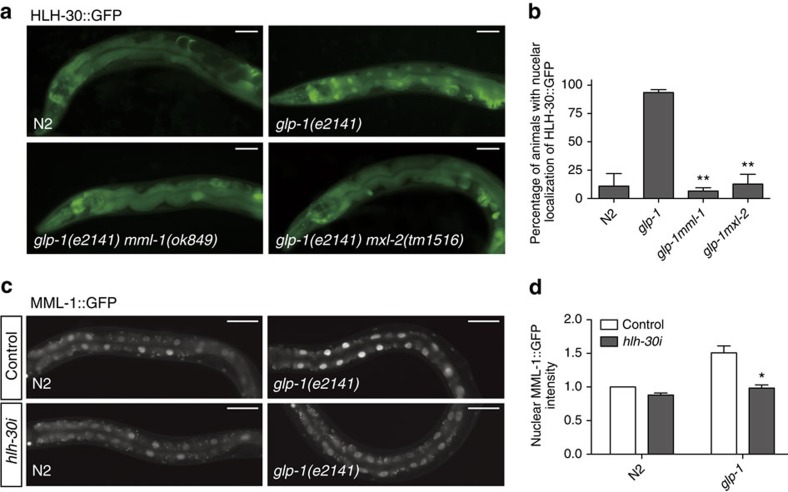
Mutual regulation of MML-1 and HLH-30. (**a**) Representative fluorescent images showing HLH-30::GFP expression in day 1 young adult worms of indicated genotypes. Worms were raised at non-permissive temperatures (25 °C) for *glp-1*. Nuclear localization of HLH-30::GFP in *glp-1* is abolished in *glp-1mml-1* and *glp-1mxl-2* animals. (**b**) Percentage of worms with HLH-30::GFP in the nuclei of intestinal cells at day 1 young adult stage (mean±s.e.m. from 3 biological replicates, >20 worms each, **P<0.01, *t*-test relative to *glp-1*). (**c**) Representative fluorescent images of MML-1::GFP transgenic worms in N2 and *glp-1* background fed with control L4440::*luc* RNAi or *hlh-30* RNAi. Upregulation of nuclear MML-1::GFP in *glp-1* is abolished on *hlh-30* knockdown. (**d**) Nuclear MML-1::GFP intensity relative to N2 fed with control RNAi (mean±s.e.m. from 3 biological replicates, >20 worms each, *P<0.05, *t*-test relative to *glp-1* fed with control RNAi). Scale bars, 50 μm.

The bHLH transcription factor HLH-30/TFEB is a conserved regulator of autophagy and lysosome biogenesis in *C. elegans* and mammals^19^,^21^,^22^. Similar to *mml-1*, it is also required for multiple forms of longevity including gonadal longevity (Supplementary Table 1). Notably, HLH-30/TFEB becomes nuclear localized in response to germline removal^19^. Given their phenotypic congruence, we asked whether there is a functional relationship between MML-1/MXL-2 and HLH-30. We found that deletion of *mml-1* or *mxl-2* dramatically impaired HLH-30::GFP nuclear accumulation in germline-less animals (Fig. 3a,b and Supplementary Fig.3a). Whereas >90% of *glp-1* animals showed HLH-30::GFP nuclear localization, <15% of *glp-1mml-1* and *glp-1mxl-2* animals evoked this phenotype. Knockdown of *mml-1* and *mxl-2* also reduced HLH-30::GFP nuclear localization in *daf-2* and *eat-2* backgrounds (Supplementary Fig. 3b,c). By contrast, *mml-1/mxl-2* had little effect on the expression of *pha-4/*FOXA (Supplementary Fig. 3d) or nuclear localization of *daf-16/*FOXO (Supplementary Fig. 3e,f), other important regulators of gonadal longevity. Moreover, *hlh-30* RNAi knockdown significantly reduced the expression levels of MML-1::GFP in germline-less animals (Fig. 3c,d and Supplementary Fig. 3g). Therefore, MML-1 and HLH-30 mutually regulate one another.

### *mml-1/mxl-2* regulates autophagy

**Figure 4:**
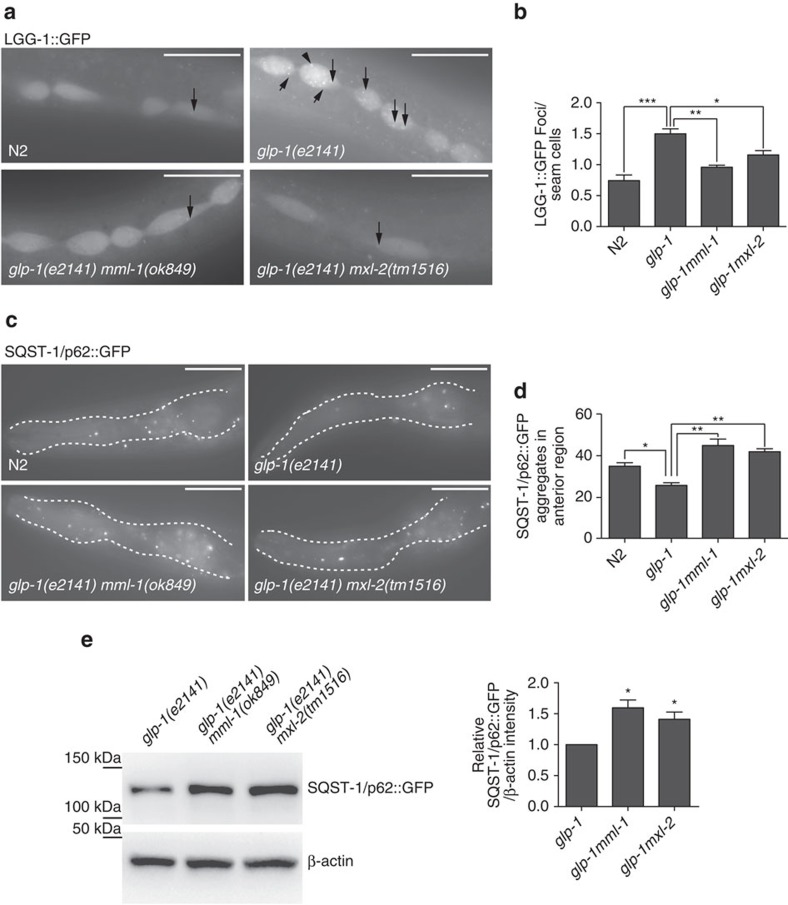
MML-1/MXL-2 promote autophagy. (**a**) Representative fluorescent images of LGG-1::GFP foci in indicated genotypes at L4 stage. LGG-1::GFP puncta are reduced in *glp-1mml-1* and *glp-1mxl-2* compared with *glp-1*. (**b**) Quantification of LGG-1::GFP puncta in seam cells. The bar represents mean±s.e.m. from 3 biological replicates (>20 worms each). *P*-value (*P<0.05, **P<0.01) relative to N2 or *glp-1* was determined by *t*-test. (**c**) Representative fluorescent images of SQST-1/p62::GFP transgenic worm of indicated genotypes at L4 stage. SQST-1::GFP aggregates are elevated in double mutants compared with *glp-1*. (**d**) Quantification of SQST-1/p62::GFP foci in the pharyngeal region. The bar represents mean±s.e.m. from 3 experiments (>20 worms each). *P*-value (**P<0.01) relative to N2 or *glp-1* was determined by *t*-test. (**e**) Representative western blotting showing SQST-1::GFP and loading control, b-actin bands in indicated genotypes (left). SQST-1::GFP is elevated in *glp-1mml-1* and *glp-1mxl-2*. Quantification of SQST-1::GFP bands is shown (right). Bar graphs show mean±s.e.m. relative to *glp-1* from three independent experiments. Band intensity of SQST-1::GFP is normalized to loading control, b-actin. *P-*value (*P<0.05) is determined by *t*-test relative to *glp-1*. Scale bars, 20 μm.

Autophagy is an essential cellular process critical to multiple longevity pathways, including gonadal longevity^14^,^16^,^17^,^18^,^20^. As HLH-30 regulates autophagy in germline-deficient animals^19^,^28^,^29^, we wondered whether MML-1/MXL-2 also affect autophagy. On autophagy induction, foci containing the LC3 homologues LGG-1 and LGG-2 become more numerous, whereas SQST-1/p62 foci are reduced. We observed that LGG-1::GFP and LGG-2::GFP puncta accumulate in the *glp-1* background and this increase depended on *mml-1(+)* and *mxl-2(+)* (Fig. 4a,b and Supplementary Fig. 4a,b). Furthermore, SQST-1::GFP foci and total protein levels were elevated in *glp-1mml-1* and *glp-1mxl-2* compared with *glp-1* (Fig. 4c–e). Previous studies showed that *hlh-30* deletion affects autophagic flux and lysosomal function, leading to the accumulation of the lysosomal marker LMP-1::GFP^19^,^28^,^29^. We observed a similar tendency in *glp-1mml-1* and *glp-1mxl-2* (Supplementary Fig. 4c). *mml-1* and *mxl-2* knockdown did not affect the basal activity of autophagy in wild-type background (Supplementary Figs. 4d–h). Altogether, these results suggest that *mml-1(+)* and *mxl-2(+)* stimulate autophagy within the gonadal longevity pathway.

### MML-1/MXL-2 interact with TOR signalling

**Figure 5:**
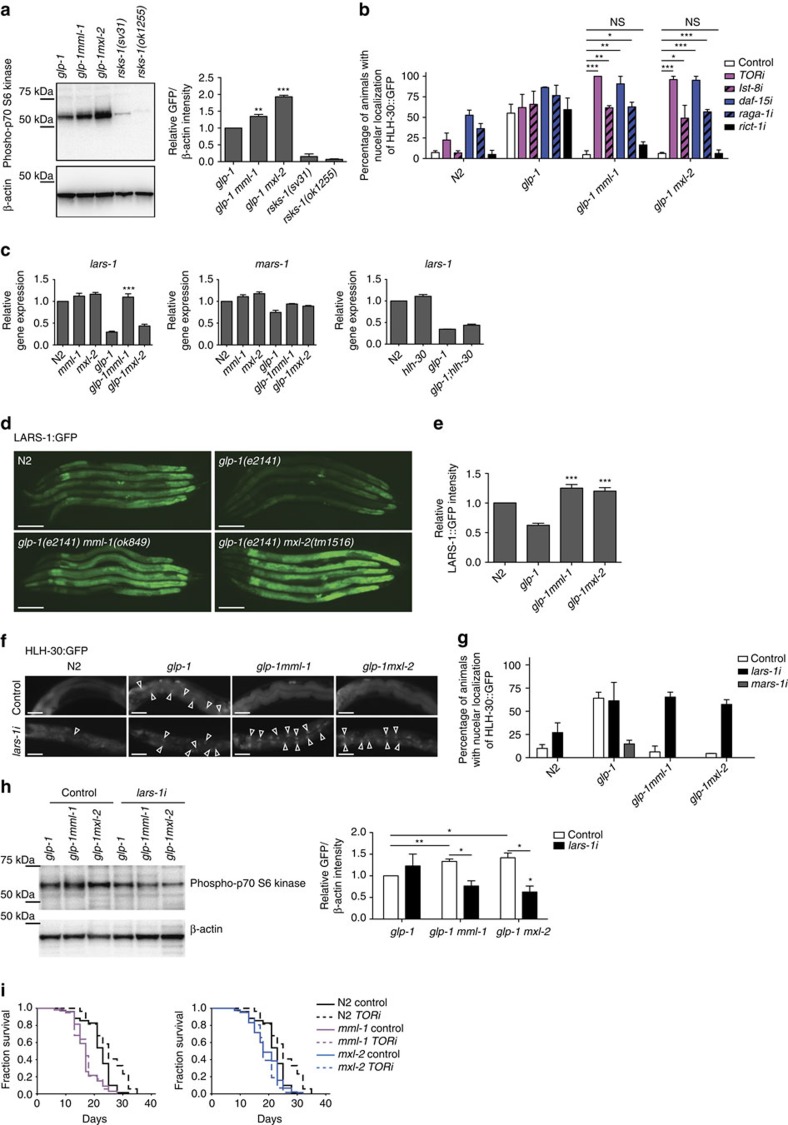
MML-1/MXL-2 regulate HLH-30 via regulating TORC1 activity. (**a**) Western blot analysis of phosphorylation of p70 S6 kinase. Bars show mean±s.e.m. of the band intensity relative to *glp-1* from three experiments. The band intensity of p70 S6 kinase phosphorylation is normalized to b-actin. *P*-value (**P<0.01 and ***P<0.001) is determined by *t*-test relative to *glp-1*. (**b**) Bars show the percentage of worms with HLH-30::GFP in the nuclei of intestinal cells at day 1 adult (mean±s.e.m. from 3 biological replicates, >20 worms each). Each RNAi treatment is indicated. Knockdown of TORC1 but not TORC2 components rescues HLH-30 nuclear localization in *glp-1mml-1* and *glp-1mxl-2*. Worms of individual genotypes were fed with indicated RNAi from egg onwards and raised at 25 °C. *P*-values (*P<0.05, **P<0.01 and ***P<0.001) were determined by *t*-test. (**c**) qRT–PCR analysis shows that *lars-1* but not *mars-1* is de-repressed in *glp-1mml-1*. The bar represents mean±s.e.m. from three biological replicates relative to N2. *P*-value (***P<0.001) was determined by one-way analysis of variance (ANOVA) with Tukey’s test relative to *glp-1*. (**d**) Representative fluorescent pictures of LARS-1::GFP. LARS-1::GFP is de-suppressed in *glp-1mml-1* and *glp-1mxl-2*. (**e**) Quantification of LARS-1::GFP in posterior intestinal cells of indicated genotypes. Bars indicate mean±s.e.m. from 3 replicates (>20 worms each). *P*-value (***P<0.001) is determined by *t*-test relative to *glp-1.* (**f**) Representative fluorescent pictures of HLH-30::GFP in indicated genotypes and RNAi treatments at day 1. *lars-1* knockdown rescues HLH-30 nuclear localization in double mutants. (**g**) Percentage of worms with HLH-30::GFP in the nuclei of intestinal cells at day 1 adult (mean±s.e.m. from 3 replicates, >20 worms each). Each RNAi treatment is indicated. (**h**) A representative western blotting showing phosphorylation of p70 S6 kinase and b-actin (left), and their quantification (right). *lars-1* knockdown by RNAi significantly reduces the level of phosphorylation of p70 S6 kinase in *glp-1mml-1* and *glp-1mxl-2* background. The bar represents mean±s.e.m. from three experiments. *P*-value (*P<0.05 and **P<0.01) was determined by *t*-test. (**i**) *mml-1* and *mxl-2* are required for TOR knockdown-mediated longevity. TOR RNAi knockdown was conducted from the day 1 adult stage. Scale bars, 100 μm (**d**) and 50 μm (**f**).

Activated TOR signalling prevents autophagy (by phosphorylation of ATG13) and promotes protein synthesis (through phosphorylation of the p70/S6 kinase and 4EBP)^30^. Recently, it has also been shown to inhibit HLH-30/TFEB nuclear localization and transcriptional activation^19^,^31^,^32^,^33^. Downregulation of TOR signalling provokes opposite effects. A simple hypothesis is that MML-1/MXL-2 regulates autophagy, HLH-30 localization and other activities via TOR signalling. To address this idea, we monitored Thr412/389 phosphorylation of S6Kinase, using anti-phosphopeptide antibodies. Consistent with hyperactivation of TOR, deletion of *mml-1* or *mxl-2* enhanced phosphorylation of S6K in the *glp-1* background (Fig. 5a) but not in the wild-type background (Supplementary Fig. 5a). Knockdown of *let-363*/TOR and TORC1-signalling components *lst8/C10H11.*8, *daf-15*/raptor and *raga-1* restored HLH-30/TFEB nuclear localization in *glp-1mml-1* and *glp-1mxl-2* backgrounds (Fig. 5b and Supplementary Fig. 5b). By contrast, knockdown of the TORC2-specific subunit *rict-1/RICTOR* had little effect. Collectively, these results indicate that MML-1/MXL-2 represses TOR activity in the gonadal longevity pathway.

### MML-1 regulates TOR signalling through LARS-1

How might MML-1/MXL-2 transcriptionally regulate TOR signalling? From RNA sequencing (RNA-seq) results (below), we found that *mml-1* regulates the leucyl-tRNA synthetase *lars-1*, whose yeast and mammalian homologues have been recently shown to stimulate TOR via RAG-GTPases^34^,^35^. *lars-1* was potently and reproducibly repressed by *mml-1(+)* in the *glp-1* background; accordingly, mutation of *mml-1* caused de-repression of the mRNA and increased levels of LARS-1::GFP (Fig. 5c–e). Data from the ModEncode project indicate that MML-1 binds directly to the *lars-1* promoter ( www.modencode.org). Similarly, LARS-1::GFP was upregulated in *glp-1mxl-2*, (although *lars-1* mRNA was not significantly regulated). By contrast, the methionyl-tRNA synthase *mars-1* was not obviously regulated in either *glp-1mml-1* or *glp-1mxl-2* (Fig. 5c). Consistent with a role in TOR signalling, we observed that *lars-1* RNAi knockdown restored HLH-30/TFEB nuclear localization in the *glp-1mml-1* and *glp-1mxl-2* backgrounds, whereas knockdown of *mars-1* had little effect (Fig. 5f,g). In addition, knockdown of *lars-1* reduced S6 kinase phosphorylation in *glp-1mml-1* and *glp-1mxl-2* backgrounds (Fig. 5h). Altogether, these observations suggest a model whereby MML-1 downregulates *lars-1* and inhibits TOR signalling. Consequently, HLH-30 becomes nuclear localized, initiating transcriptional cascades.

Despite the restoration of HLH-30 nuclear localization, knockdown of *let-363/*TOR, surprisingly, failed to rescue longevity in the *mml-1* and *mxl-2* background (Fig. 5i). Furthermore, *let-363*/TOR knockdown failed to fully restore autophagy or longevity to *glp-1mml-1* and *glp-1mxl-2* double mutants (Supplementary Fig. 5c,d), nor did *hlh-30(+)* overexpression or *raga-1* deletion rescue *glp-1mml-1* and/or *glp-1mxl-2* short-lived phenotypes (Supplementary Fig. 5e and Supplementary Table 1). These results suggest that MML-1 does not work in a strict linear pathway and may act parallel to or downstream of TOR signalling to mediate additional outputs. By inference, HLH-30 nuclear localization is not sufficient for longevity in this context and MML-1 may promote other key life-extending activities.

### Shared and preferential targets of MML-1/MXL-2 and HLH-30

To gain further insight into activities of these transcription factors, we compared transcriptomes of wild type, *glp-1*, *mml-1*, *mxl-2*, *hlh-30* and double mutants with *glp-1* for differentially expressed genes (DEGs) measured by RNA-seq. We used a false discovery rate of 5% when considering DEGs. Hierarchical clustering of gene expression values revealed that biological replicates clustered well together, demonstrating the sample quality (Supplementary Fig. 6a).

**Figure 6:**
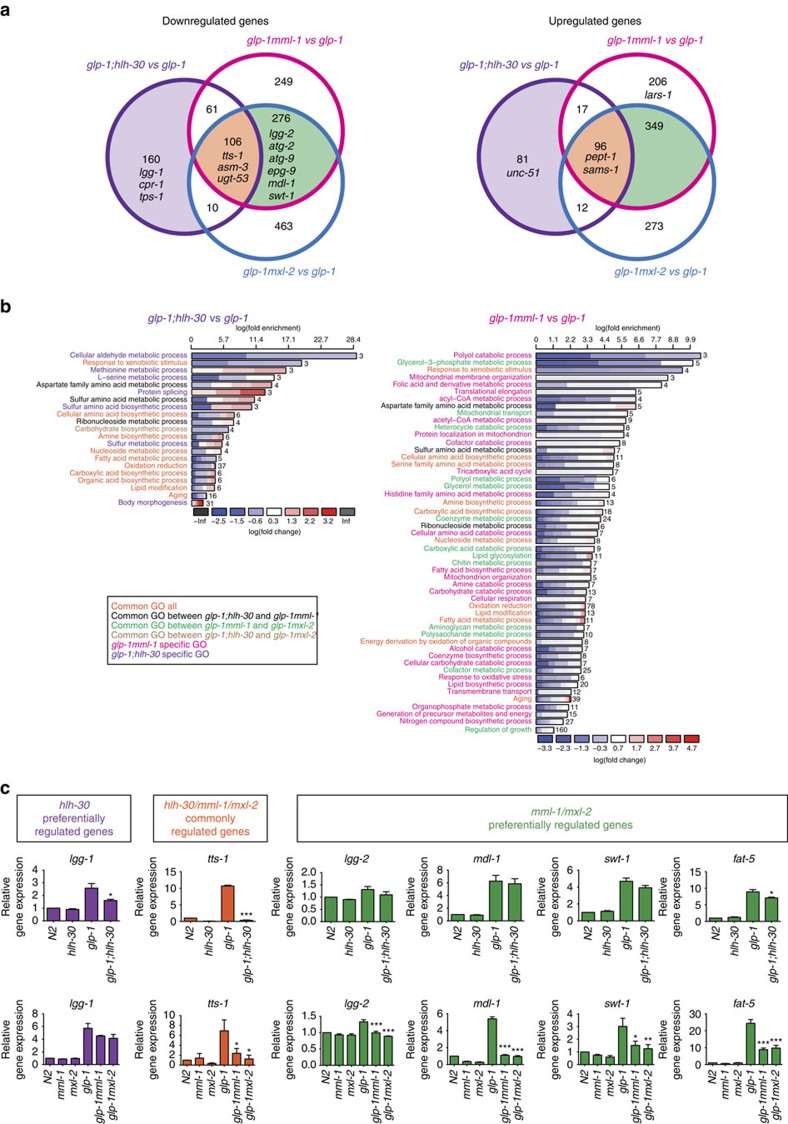
Transcriptome analysis reveals preferentially regulated and commonly regulated genes by MML-1/MXL-2 and HLH-30. (**a**) Venn diagrams depict the numbers of DEGs identified through RNA-seq analysis from *glp-1mml-1* versus *glp-1*, *glp-1mxl-2* versus *glp-1* and *glp-1;hlh-30* versus *glp-1*. (**b**) GO enrichment analysis using DAVID. The enrichment (upper *x* axis), fold change (colour coding) and number of genes are shown (next to each bar). See Supplementary Data 5 for details. (**c**) qRT–PCR analysis confirms *hlh-30* preferentially regulated genes (*lgg-1*), *hlh-30/mml-1/mxl-2* commonly regulated genes (*tts-1*) and *mml-1/mxl-2* preferentially regulated genes (*lgg-2*, *mdl-1*, *swt-1* and *fat-5*). The bar represents mean±s.e.m. from three biological replicates relative to N2. *P*-value (* *P*<0.05, **P<0.01 and ***P<0.001) was determined by one-way analysis of variance (ANOVA) with Tukey’s test relative to *glp-1*.

*mml-1* or *mxl-2* deletion resulted in numerous DEGs that were commonly regulated in either wild type or *glp-1*, including both up- and downregulated genes (Fig. 6a, Supplementary Fig. 6b and Supplementary Table 4). For example, 827 genes (382 downregulated and 445 upregulated) were commonly regulated by *mml-1* and *mxl-2* in the *glp-1* background. Each transcription factor also regulated large gene sets independent of the other, in particular *mxl-2* deletion, which resulted in hundreds of genes being regulated differently from *mml-1*.

Gene Ontology (GO) term analysis of *glp-1mml-1* versus *glp-1* revealed an enrichment of genes implicated in oxidation/reduction, ageing, lipid modification, carbohydrate catabolic process, mitochondrial transport and amine biosynthetic pathway among others (Fig. 6b and Supplementary Data 1). Kyoto Encyclopedia of Genes and Genomes (KEGG) process analysis also revealed enrichment in lysosomal function and fatty acid desaturation (Supplementary Data 2). *mxl-2*-dependent DEG in the *glp-1* background included similar GO terms as *glp-1mml-1* but also included GO terms specific to *mxl-2* such as oocyte development, β-oxidation, regulation of translation and cell division among others (Supplementary Fig. 6c and Supplementary Data 1).

When we manually interrogated our RNA-seq data, we found several genes regulated by *mml-1* and *mxl-2* to be involved in autophagy including *lgg-2*, *atg-2*, *atg-9* and *epg-9*, and in lysosomal function including *ctns-1*, *cpr-3* and *cpr-5*, many of which we confirmed by quantitative PCR (qPCR) (Fig. 6c and Supplementary Fig. 6d). We also found worm homologues of MondoA and ChREBP transcriptional targets involved in glucose and lipid metabolism to be significantly regulated in *mml-1*, including the glucose transporter *fgt-1*, pyruvate kinase *pyk-1*, glycerol 3-phosphate dehydrogenases *gpdh-1*, the SREBP homologue *sbp-1* and steroyl fatty acid desaturase homologue *fat-5*, some of which we confirmed by qPCR (Fig. 6c and Supplementary Fig. 6d).

A comparison of the MML-1/MXL-2 and HLH-30 transcriptomes in the *glp-1* background (Fig. 6a) revealed at least three sets of interest: the ‘*hlh-30/mml-1/mxl-2* commonly regulated genes’, ‘*hlh-30* preferentially regulated genes’ and ‘*mml-1/mxl-2* preferentially regulated genes’. (preferentially regulated means that one or the other transcription factor predominately but not exclusively regulates the gene of interest). As predicted, HLH-30 and MML-1/MXL-2 regulated a substantial number of genes in common (106 genes downregulated, 96 genes upregulated, and 28 genes differentially regulated), supporting the idea that they work in overlapping transcriptional cascades. Processes regulated in common to all genotypes included metabolism of xenobiotics, amino acids, fatty acids, amines and nucleosides, as well as oxidation/reduction and ageing (Fig. 6b). Commonly regulated genes included *tts-1*, *sams-1 pept-1*, *asm-3* and *ugt-53* (Fig. 6c and Supplementary Fig. 6f), most of which affect longevity^36^,^37^,^38^ (Supplementary Fig. 6g). Although both *hlh-30* and *mml-1/mxl-2* regulated autophagy, they preferentially controlled distinct gene sets: *hlh-30* predominately regulated *lgg-1/atg-8* and *unc-51/atg-1*, whereas *mml-1/mxl-2* predominately regulated *atg-2*, *lgg-2*, *atg-9* and *epg-9* (Fig. 6c and Supplementary Fig. 6d,e).

### TOR regulates gene expression via a HLH network

**Figure 7:**
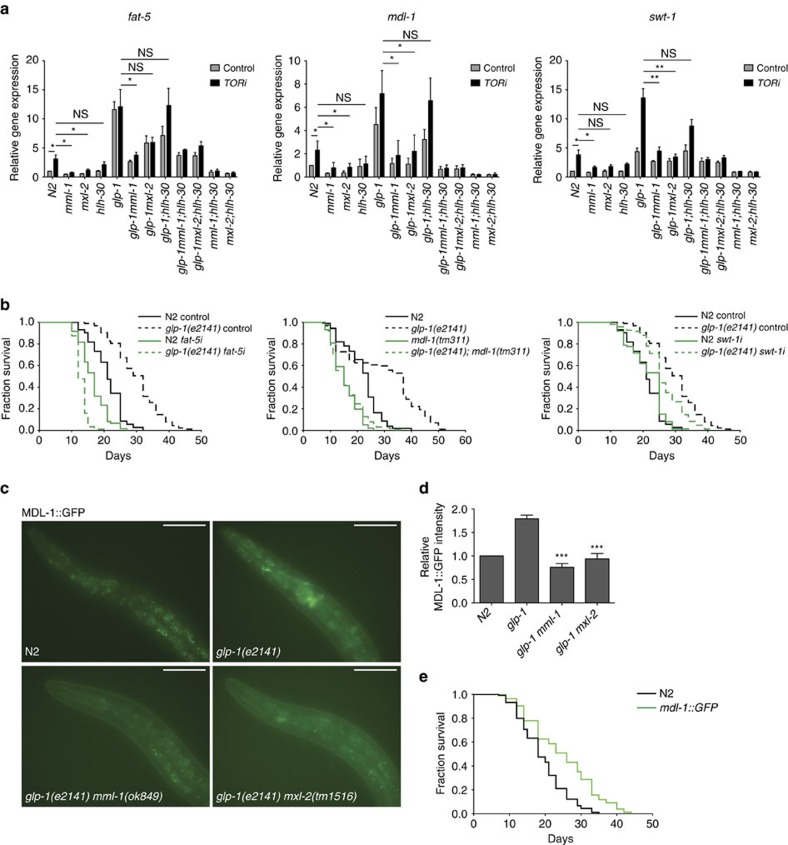
MML-1/MXL-2 preferentially regulated genes contribute to gonadal longevity. (**a**) qRT–PCR analysis of MML-1/MXL-2 preferentially regulated genes after treatment with control or TOR RNAi. The bar represents mean±s.e.m. from three biological replicates relative to N2. *P*-value (*P<0.05 and **P<0.01) was determined by one-way analysis of variance (ANOVA) with Tukey’s test. (**b**) Knockdown of *fat-5*, *mdl-1* and *swt-1* significantly suppress *glp-1* longevity. (**c**) Representative fluorescent images of MDL-1::GFP in indicated strains at day 1 adult. Upregulation of MDL-1::GFP in *glp-1* background is abolished by *mml-1* or *mxl-2* deletion. (**d**) Quantification of MDL-1::GFP in the head region in indicated genotypes. Bars indicate mean±s.e.m. from 3 biological replicates (>20 worms each). *P*-value (***P<0.001) was determined by *t*-test. (**e**) MDL-1 overexpression extends wild-type lifespan. Scale bars, 50 μm (**c**).

We next wondered how TOR perturbation would affect the expression of representative genes from the three categories. First, TOR knockdown in the wild type or *glp-1* background induced expression of genes such as *fat-5*, *swt-1* and *mdl-1*, which showed preferential dependence on *mml-1* and *mxl-2*, and less dependence on *hlh-30* (Fig. 7a). Second, TOR knockdown triggered *tts-1* expression in wild type and *glp-1*, which was largely dependent on *hlh-30* and less dependent on *mml-1*/*mxl-2* (Supplementary Fig. 7a). Third, a handful of *mml-1/mxl-2* preferentially regulated autophagy genes such as *atg-2* and *lgg-2* were rescued by TOR knockdown in an *hlh-30*-dependent manner, suggesting that HLH-30 can compensate (Supplementary Fig. 7a). Relatedly, double mutation of *hlh-30* and *mml-1* in many cases (for example, *atg-2* and *lgg-2*) abolished TOR-regulated gene expression. Finally, *sams-1*, a commonly regulated gene, was largely rescued by TOR knockdown in double- and triple-mutant backgrounds (Supplementary Fig. 7a), suggesting that yet other factors can functionally substitute. Taken together, our results suggest that TOR knockdown triggers transcriptional responses through *mml-1*, *mxl-2*, *hlh-30* and other factors, revealing that these HLH transcription factors are major mediators of TOR signalling.

### MML-1/MXL-2 regulated genes affect gonadal longevity

We reasoned that genes preferentially regulated by *mml-1*/*mxl-2* should contribute to *mml-1*-induced longevity. Focusing on the *mml-1*-specific DEGs, we knocked down representative *ca*. 120 genes that showed upregulation in an *mml-1*-dependent manner. A majority of these knockdowns reduced survivorship of *glp-1* mutants by up to 30%. Among this set were genes previously implicated in longevity, such as *mdl-1* (refs 26, 39), as well novel candidates *swt-1* and *fat-5.* We confirmed that knockdown of these candidates significantly reduced *glp-1* longevity in demography experiments (Fig. 7b and Supplementary Fig. 7b). Interestingly, we found that MDL-1::GFP was upregulated in germline-deficient animals in a *mml-1/mxl-2*-dependent manner (Fig. 7c,d). This upregulation contributes to longevity, as *mdl-1* overexpression extended wild-type lifespan (Fig. 7e) and stimulated expression of specific autophagy genes (Supplementary Fig. 7c). Consistent with a role in autophagy, *mdl-1* knockdown decreased the number of LGG-1::GFP foci and increased SQST-1/p62::GFP foci in *glp-1* background (Supplementary Fig. 7d–f). It is interesting to note that *mdl-1* knockdown further decreased LGG-1::GFP foci even in *glp-1mml-1* and *glp-1mxl-2* background, suggesting that *mdl-1* additionally regulates autophagy downstream of *mml-1/mxl-2*. We also found that *mdl-1* overexpression partially rescued the short-lived phenotype of *glp-1mml-1* and *glp-1mxl-2* (Supplementary Fig. 7g). Moreover, the *mdl-1* heterodimeric partner *mxl-1* was required for *glp-1* longevity (Supplementary Fig. 7h). These findings support the idea that *mml-1/mxl-2* can regulate targets somewhat independently of HLH-30 to affect lifespan and indicate that *C. elegans* Myc/Mondo family members work in an expansive cascade to regulate gonadal longevity.

### MondoA and ChREBP regulate TFEB nuclear localization

**Figure 8:**
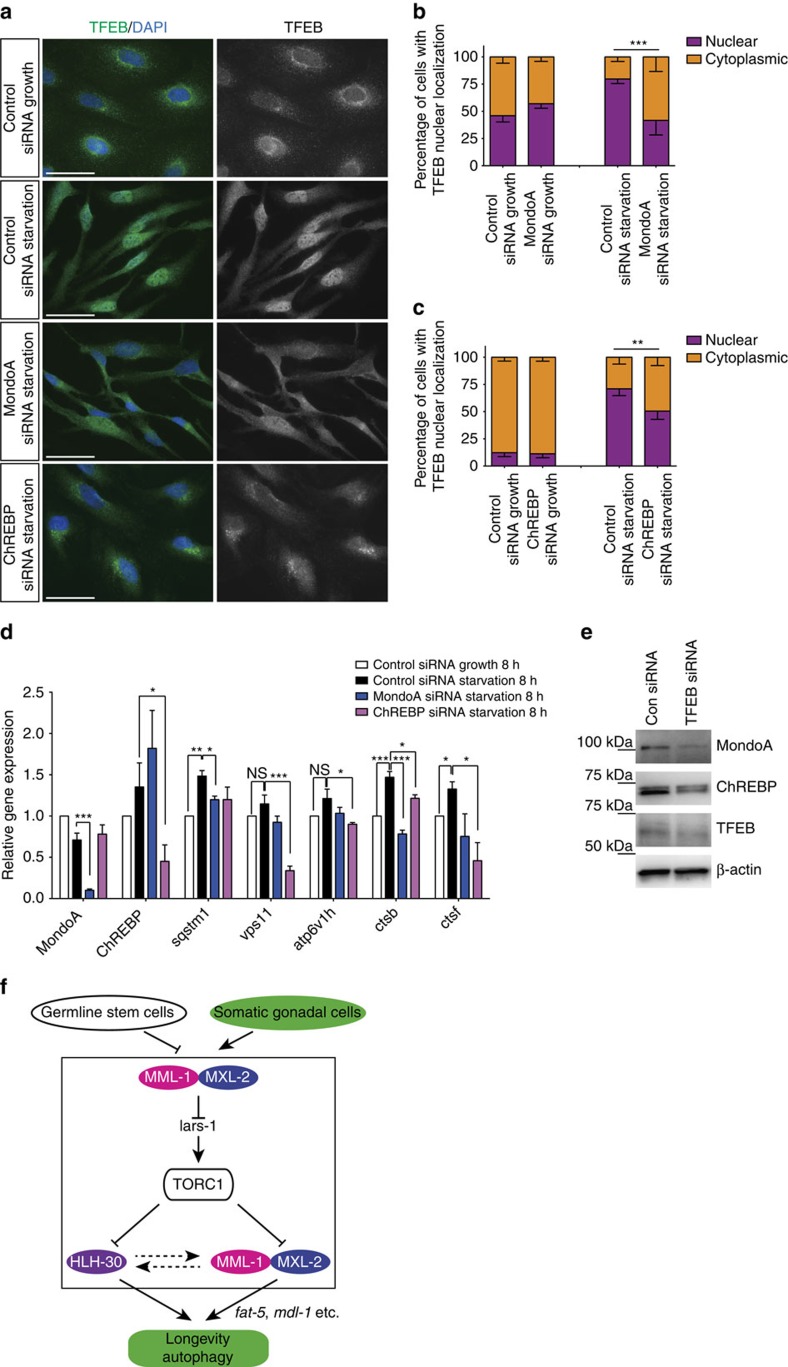
MondoA and ChREBP knockdown compromise TFEB nuclear localization and target gene expression. (**a**) Representative fluorescent images of TFEB nuclear localization on control, MondoA or ChREBP knockdown in HeLa cells. Starvation-induced TFEB nuclear localization (4 h) is impaired by MondoA and ChREBP siRNA knockdown. (**b**) Quantification of TFEB nuclear localization on control or MondoA knockdown. (**c**) Quantification of TFEB nuclear localization on control or ChREBP knockdown. Bars indicate mean±s.e.m. from four biological replicates. *χ*^2^-test, **P<0.01 ***P<0.001 (**b**,**c**) (**d**) TFEB target gene expression is affected on MondoA and ChREBP knockdown. The bar represents mean±s.e.m. from five experiments relative to control siRNA 8-h growth condition. *P*-value (*P<0.05 and ***P<0.001) was determined by one-way analysis of variance (ANOVA) with Tukey’s test relative to control siRNA growth or starvation condition. (**e**) Representative western blotting showing that TFEB knockdown leads to decreased steady-state levels of MondoA and ChREBP. (**f**) A working model based on the current study. MML-1 is upregulated on germline removal. Activated MML-1/MXL-2 represses TOR activity by downregulating *lars-1*, leading to the nuclear localization of HLH-30. MML-1/MXL-2 also work downstream of TOR and activate preferential targets such as MDL-1, to regulate autophagy and longevity. HLH-30 also upregulates MML-1 through an unknown mechanism (see also Discussion). Scale bars, 50 μm (**a**).

We next asked whether the regulatory cascades observed in *C. elegans* are conserved in mammals. To test whether MondoA or ChREBP (MondoB) regulate TFEB, we analysed expression and localization in HeLa cells on gene knockdown. First, as observed previously^31^,^32^, amino acid starvation enhanced endogenous TFEB nuclear localization (Fig. 8a). When cells were exposed to MondoA small interfering RNA (siRNA), TFEB nuclear localization significantly diminished (control starvation, 79.6±4.1% versus MondoA starvation, 41.6±13.3%; Fig. 8a,b and Supplementary Fig. 8a). ChREBP siRNA knockdown also modestly affected TFEB nuclear localization (three of four experiments) (control starvation, 71.0±6.2% versus ChREBP starvation, 50.5±7.6%; Fig. 8a,c and Supplementary Fig. 8b). Correlatively, TFEB direct target genes showed reduced expression, although surprisingly, with some specificity: MondoA knockdown more dramatically affected *ctsb*, whereas ChREBP knockdown blunted the expression of *vps11* (Fig. 8d and Supplementary Fig. 8c). Finally, TFEB knockdown led to decreased steady state levels of MondoA and ChREBP in cell culture (Fig. 8e). These findings indicate that mammalian MondoA/ChREBP and TFEB mutually regulate one another.

## Discussion

Numerous transcriptional factors mediate the outputs of the major longevity pathways^1^; yet, how they form coherent transcriptional regulatory networks is not understood. The gonadal longevity pathway is a rich multi-layered regulatory network, reflecting the intense evolutionary pressure to propagate an intact germline in the face of changing environments, nutrient conditions and physiologic milieus. In this work, we unravel a core regulatory node governing *C. elegans* gonadal longevity. We have found that the HLH Mondo/Max-like complex MML-1/MXL2 is required to promote longevity of germline-less animals. This complex also promotes longevity triggered by reduced TOR and insulin/IGF signalling, reduced mitochondrial respiration in some measure, as well as reportedly by dietary restriction^26^. Thus, MML-1/MXL-2 is among a handful of select factors working at the convergence of major longevity pathways.

Most interestingly, we have discovered that the MML-1/MXL-2 complex dramatically stimulates nuclear localization and activity of the TFEB homologue HLH-30, a convergent regulator of autophagy, lysosome biogenesis, fat metabolism and longevity^19^,^28^,^29^, via TOR signalling. In germline-less animals, MML-1/MXL-2 transcriptionally repress the leucyl-tRNA synthase LARS-1, a positive effector of the TOR signalling pathway^34^,^35^, which results in HLH-30 nuclear localization and activity (see Model; Fig. 8f). HLH-30 itself promotes upregulation of MML-1 in the germline-less *glp-1* background. Our findings expand the functional outputs of MML-1/MXL-2 complex as key regulators of autophagy, TOR and TFEB signalling. Importantly, we found that aspects of these regulatory relationships are evolutionarily conserved: mammalian homologues MondoA and ChREBP stimulate TFEB nuclear localization on amino acid starvation in cell culture. Thus, MML-1/Mondo and HLH-30/TFEB form a tight regulatory ensemble linked through TOR signalling, as well as presumably through E-box elements commonly bound by HLH transcription factors.

bHLH transcription factors regulate proliferation, growth, development and metabolism, and form an extensive interlocking network through heterodimerization and similar DNA-binding sites. The *C. elegans* HLH transcription factors have been assembled into an incipient network and Myc/Mondo complexes bind to similar E-box elements (CACGTG) as HLH-30/TFEB^22^,^23^,^40^. Consistent with interlinked regulatory cascades, our transcriptome data indicate that MML-1, MXL-2 and HLH-30 substantially overlap in target gene expression, but also have preferential outputs. MML-1 and MXL-2 are well known to work in a complex^23^,^40^ and accordingly show considerable overlap in their transcriptomes, tissue distribution and their mutant phenotypes. Surprisingly, more genes were upregulated than downregulated in the mutants, suggesting that this ‘activation complex’ may well have potent repressive activity. MXL-2 shows broader transcriptomic changes than MML-1, indicating participation in other complexes. Supporting the idea of independence, *mml-1* and *mxl-2* mutants exhibit somewhat different patterns of genetic epistasis, for example, *mml-1* is required for *isp-1* longevity, whereas *mxl-2* is not. Perhaps this difference reflects our observed association of MML-1 with the mitochondria and the enrichment of mitochondria GO terms from *mml-1* transcriptome analysis.

MML-1 and HLH-30 also show significant transcriptional overlap but preferentially regulate distinct gene sets. Overlapping-regulated processes include amino acid, fat, amine, nucleoside and xenobiotic metabolism, oxidation/reduction and others, possibly pinpointing processes important for longevity (Fig. 6). Interestingly, sulfur amino acid metabolism has been linked to methionine restriction and H_2_S metabolism, which affect life span^41^. Another common striking output is *tts-1*, a long non-coding RNA that represses protein synthesis and affects multiple longevity pathways^36^,^37^,^38^. As *mml-1* and *hlh-30* represent convergent regulators of longevity, further functional dissection of their transcriptomes should illuminate core processes for lifespan extension. ModEncode data indicate that MML-1 and HLH-30 often reside at the same promoters^23^,^40^,^42^. Currently, it is not known whether the two factors bind independently or cooperatively, act in series as part of transcriptional cascades or work together in a complex. In addition, their temporal dynamics and whether they function in a tissue- or stage-specific manner remain to be elucidated. Future work to identify transcriptional complexes and their binding sites in the genome (chromatin immunoprecipitation sequencing) in the context of ageing should clarify these issues.

Although HLH-30 overexpression modestly extends lifespan^19^, our data indicate that HLH-30 activity is not sufficient to promote longevity in the gonadal pathway. Although knockdown of TOR signalling partially bypassed the *mml-1/mxl-2* requirement for HLH-30 nuclear localization and activity in the *glp-1* background, it was, surprisingly, unable to drive autophagy and longevity. This observation indicates that these factors act within partially overlapping networks, rather than a strict linear pathway, and that *mml-1/mxl-2* leverages key processes for longevity. Accordingly, *mml-1* preferential transcriptional targets *fat-5*, *swt-1* and *mdl-1* also facilitated longevity. Previously, Goudeau *et al*.^13^ showed that *fat-5* deletion does not affect *glp-1* longevity, which is contradictory to our results (Fig. 7b and supplementary Fig. 7b). Although the exact cause for this difference is unknown, Goudeau *et al*.^13^ used bacterial strain HT115, whereas we used OP50, suggesting that the food source may be responsible. *mml-1* and *hlh-30* also regulated the process of autophagy in common but preferentially affected distinct components: *mml-1* mainly affected *atg-2* and *lgg-2*, whereas *hlh-30* affected *atg-1* and *lgg-1.* Conceivably, other *mml-1/mxl-2*-enriched processes such as glycerol or mitochondrial metabolism also contribute.

Our data suggest that MML-1 represents an ancestral MondoA/ChREBP transcription factor conserved at many levels, which can inform mammalian metabolism and ageing. First, MML-1 and Mondo share common protein-domain structure including nuclear localization and glucose regulatory domains at the amino terminus and the bHLH-Zip domains at the carboxy terminus^23^. Second, *mml-1* target genes are remarkably similar to those regulated by mammalian counterparts including genes involved in sugar transport (*fgt-1*), fatty acid desaturation (*fat-5*), pyruvate kinase (*pyk-1*), sterol regulatory binding protein (*sbp-1*) and glycerol-3-phosphate dehydrogenase (*gpdh-1*)^24^,^27^,^43^. Whether *C. elegans* MML-1 senses glucose metabolites and has an impact on glycolysis and lipogenesis remain to be tested. Conversely, novel genes and processes regulated by *C. elegans* MML-1/MXL-2 (for example, autophagy, lysosomal and mitochondrial function) are plausible targets to further test in the mammalian system.

Third, our work here reveals an evolutionarily conserved interrelationship between MONDO and TFEB. In response to germline removal, MML-1/MXL-2 promotes HLH-30 nuclear localization and activation; conversely, HLH-30 also upregulates MML-1 in germline-less animals. Similarly, MondoA and ChREBP facilitate TFEB nuclear localization and gene expression in response to amino acid starvation in cell culture, and TFEB knockdown reduced MondoA and ChREBP expression, revealing that the homologous transcription factors show mutual regulation. MondoA and ChREBP furthermore regulated TFEB gene targets, revealing a novel role in lysosomal function but, unexpectedly, affected distinct genes, perhaps reflecting their functional or temporal diversification. Other transcription factors working within the gonadal pathway show dependence: DAF-12/steroid receptor signalling facilitates DAF-16/FOXO nuclear localization^44^,^45^ and MXL-2 stabilizes MML-1 (ref. 23). Speculatively, such positive reinforcement could stabilize a fragile interdependent network architecture to maintain coherent states geared towards reproduction or survival.

Fourth, MML-1/MXL-2 and MondoA/ChREBP function are intimately linked to TOR signalling. On the one hand, our data suggest that MML-1 suppresses TOR in germline-less animals; *mml-1* loss-of-function hyperactivates TOR through upregulation of *lars-1* and perhaps other factors, resulting in increased S6K activity, decreased autophagy and impaired nuclear localization of HLH-30. On the other hand, *mml-1* loss often suppressed transcriptional changes and longevity arising from TOR knockdown. Thus, MML-1 may act both upstream, to transcriptionally repress TOR signalling, and downstream, to mediate the output of reduced TOR signalling. By inference, MML-1 may work in a feedback loop to limit TOR signalling. Transcription factors genetically regulated by *C. elegans* TOR are limited to DAF-16/FOXO, SKN-1/NFE2, PHA-4/FOXA and HLH-30/TFEB^19^,^46^,^47^, but mechanistic links still remain obscure. Thus, the identification of MML-1/MXL-2 as potent suppressors of TOR-induced autophagy, longevity and gene expression reveals these transcription factors as critical outputs of this nutrient-sensing pathway.

Although MondoA and ChREBP are best known as glucose sensors that signal replete states, our studies suggest a potential role under nutrient limitation. Accordingly, Ayer and colleagues^48^ have recently shown that mammalian MondoA suppresses mammalian TOR (mTOR) through upregulation of the TXNIP (thioredoxin-interacting protein), a regulator of glucose uptake and cellular redox signalling. Conversely, mTOR binds and sequesters MondoA activity in response to reactive oxygen species (ROS) and mTOR knockdown stimulates specific MondoA gene targets. MondoB/ChREBP also binds to mTOR. Whether MML-1/MondoA/ChREBP are direct targets of TOR phosphorylation is currently unknown. It is also unclear how germline removal triggers MML-1 activation: is it through glucose or related metabolites, TOR, ROS signalling or other signals? In this regard, it is intriguing that both MML-1 and MondoA are not only nuclear localized but are also present in the mitochondria, a site of ROS production^27^. Preliminary data also suggest that a fraction of MML-1 resides at the lysosome (S.N. and A.A., unpublished observation), a critical site of TOR action and TFEB localization^31^,^33^.

Finally, our studies highlight a convergent core role for Myc/Mondo complexes in the regulation of animal longevity. Previous work implicated *mml-1/mxl-2* in IIS and dietary restriction pathways^26^; our studies greatly expand this role to longevity induced by HLH-30, germline loss, decreased TOR signalling and, in part, mitochondrial longevity. In recent times, Sedivy and colleagues^49^ showed that Myc +/− heterozygote mice exhibit healthier lipid metabolism, increased physical activity, higher metabolic rate, less age-related pathologies and enhanced longevity. Molecular correlates include reduced IGF, increased AMPK, decreased AKT, mTOR and S6K activity, and protein synthesis. Evidently, various members of the Myc/Mondo HLH transcription factor family function to regulate metazoan lifespan through major growth factor and energy- and nutrient-sensing pathways. It is striking that among target genes regulated by MML-1/MXL-2 is yet another member of the Myc subfamily, MDL-1, encoding the Mad-like homologue, which we found to be required for germline-less longevity, and whose overexpression is sufficient to extend life. We therefore suggest that Myc/Mondo HLH transcription factors comprise an extensive cascade regulating metabolism and longevity. Disentangling their distributed and shared responsibilities, as well as cross-regulation, and understanding how these interactions specifically impact metabolism and ageing will be fascinating to explore.

## Methods

### *C. elegans* growth conditions

Nematodes were cultured using standard techniques at 20 °C on nematode growth medium agar plates with the *Escherichia coli* strain OP50, unless otherwise noted. Strains with *glp-1(e2141)* background were maintained at 15 *coli* °C and grown at 25 °C to induce germline-deficient phenotype. All strains used in this study are listed in Supplementary Table 2.

### Lifespan assays

Synchronized eggs were obtained by 4–6 *coli*h egg lay. When they reached day 1 adults, lifespan experiments were set up at a density of 15–20 animals per plate and carried out at 20 °C. These animals were transferred to new plates every other day. Survivorships of worms were also counted every other day. Death was scored as the absence of any movement after stimulation by a platinum wire. For lifespan experiments using *isp-1(qm150)* strains, egg lays were conducted 3 days before that of other strains, to obtain day 1 adults at the same time. Worms undergoing internal hatching, bursting vulva or crawling off the plates were censored. For all experiments, strains and/or conditions were blinded. For the ablation of germline precursor cells, newly hatched L1 worms were anaesthetized in 0.5% phenoxypropanol in M9 buffer. Z2/Z3 germline precursor cells were ablated by laser microsurgery (Micropoint) and successful ablations were confirmed under the dissecting microscope at day1 adult stage. The controls (mock ablated) were also anaesthetized in parallel with experimental animals and included for lifespan assay. For lifespan experiments using the *glp-1* strain, all strains were raised at 25 °C for 2 days, to induce the *glp-1* phenotype after the egg lay at 15 °C. Subsequently, lifespan experiments were resumed at 20 °C. In some cases, we observed a relatively long median lifespan of N2 under these conditions, presumably due to hormesis effects of the temperature shift. For all lifespan experiments, strains and/or conditions were blinded. Statistical analyses were performed with the Mantel–Cox log rank method in Excel (Microsoft).

### RNA interference

RNAi was conducted by feeding HT115 (DE3) bacteria transformed with L4440 vector that produces double-stranded RNA against the targeted gene. Synchronized eggs obtained by egg lay were put on corresponding RNAi plates containing isopropyl-β-D-thiogalactoside and ampicillin. RNAi clones were available from the Ahringer or Vidal RNAi library. *let-363/TOR* RNAi clone was a gift from Dr Hansen (Sanford-Burnham Medical Research Institute). Empty vector (L4440) or Luciferase (L4440::Luc) RNAi were used as non-targeting control. *glp-1(e2141)* suppressor screening was conducted using transcription factor RNAi library^7^. *glp-1(e2141)* worms were synchronized by tight egg lay and treated by control RNAi and RNAi targeting transcription factors. The survivorship at day 25 was used as an estimate of longevity. RNAi suppressor screening was replicated twice and only the candidates that show reproducible results were taken into consideration for further analysis. For all genes that are targeted by RNAi in this study, we indicated ‘i’ after the gene name.

### Plasmid construction and transgenesis

*mml-1*::*GFP* translational fusion construct that contains 4-kb promoter and *mml-1* coding sequence is a kind gift from Dr. Ayer^23^. For *mxl-2*::*GFP* and *lars-1*::*GFP* translational fusion constructs, *mxl-2* and *lars-1* 4-kb endogenous promoter plus coding sequence were cloned into pDC4 vector, which contain an enhanced green fluorescent protein (GFP) tag, respectively. Microinjection of these constructs was carried with the co-injection marker *myo-2p*::*mCherry* to generate *dhEx966* (*mml-1p::mml-1::GFP*, *myo-2p::mCherry*), *dhEx967* (*mml-1p::mml-1::GFP*, *myo-2p::mCherry*), *dhEx1037* (*mxl-2p::mxl-2::GFP*, *myo-2p::mCherry*) and *dhEx1031* (*lars-1p::lars-1::GFP*, *myo-2p::mCherry*). We obtained *mdl-1*::GFP from the CGC and outcrossed the strain with our wild type, to use for lifespan and microscopy experiments. *mdl-1*::GFP strain, *unc-119(ed3) III; wgIs106[mdl-1::TY1::EGFP::3xFLAG+unc-119(+)]*, which contains the endogenous *mdl-1* promoter and coding region, was originally made by TransgeneOme project (https://transgeneome.mpi-cbg.de/transgeneomics/index.html).

### RNA extraction and qRT–PCR

Worms and cell samples were harvested in TRIzol (Invitrogen). Total RNA was extracted using RNeasy or miRNAeasy kit (QIAGEN). Complementary DNA was generated using iScript (Bio-Rad). Quantitative reverse transcriptase–PCR (qRT–PCR) was performed with *Power* SYBER Green (Applied Biosystems) on a ViiA 7 Real-Time PCR System (Applied Biosystems). Four technical replicates were performed in each reaction. *ama-1* and/or *cdc-42* (for worms), and GAPDH (for cells) were used as an internal control. Primer sequences are listed in Supplementary Table 3.

### Western blotting

Worms at L4 or day 1 adult were lysed in worm lysis buffer (50 mM Tris-HCl pH 7.4, 150 mM NaCl, 1 mM EDTA, 0.1% NP-40, protease and phosphatase inhibitor cocktail (Roche)) using a dounce homogenizer.^50^ After the centrifugation, the supernatants were used for protein measurement and subsequent western blotting analysis. Worm protein lysates were separated by SDS–PAGE (Bio-Rad) and transferred to polyvinylidene difluoride membrane (Bio-Rad). Membranes were incubated with specific primary antibodies. Primary antibodies and dilutions used for worm western blotting are as follows: mouse anti-GFP (1/2,500, Clontech, 632380), rabbit anti-phospho-p70 S6 kinase (Thr412) (1/500, Millipore, 07-018), which detects conserved residue Thr404 in worms, and mouse anti-b-actin (1/10,000, Abcam, ab8224). The band intensity was quantified by Image J (version 1.48) (http://imagej.nih.gov/ij/). The uncropped western blotting results are shown in Supplementary Fig. 9.

### Microscopy and quantification of GFP

To monitor the autophagy activity, LGG-1::GFP, SQST-1/p62::GFP and LGG-2::GFP animals were anaesthetized in 0.1% sodium azide and GFP puncta in the seam cells (LGG-1::GFP), in the pharyngeal region (SQST-1/p62::GFP) or in the tail region (LGG-2::GFP) were counted at L4 stage after taking pictures using a Zeiss Axio Imager Z1 microscope. For quantification of MML-1::GFP, MXL-2::GFP, HLH-30::GFP, DAF-16::GFP and MDL-1::GFP, animals were anaesthetized in 1 mM Levamisole or 0.1% sodium azide and pictures were taken at day 1 adult stage at the same exposure time using a Zeiss Axio Imager Z1 microscope. GFP intensity in the intestine (MML-1::GFP, MXL-2::GFP, HLH-30::GFP and DAF-16::GFP) or the head region (MDL-1::GFP) was measured and quantified using Image J (version 1.48). For LARS-1::GFP quantification, animals were aligned on pre-cooled unseeded plates and pictures were taken at day 1 adult stage using Leica M165 FC microscopy. LARS-1::GFP intensity of posterior intestine was measured and quantified using image J (version 1.48). For the nuclear localization assay of HLH-30::GFP and MML-1::GFP, animals were anaesthetized in 0.1% sodium azide at day 1 adult stage and the numbers of animals carrying nuclear HLH-30::GFP or MML-1::GFP were quantified. For all microscopy experiments, more than 20 worms were scored in each experiment and all experiments were repeated at least three times.

### Mitochondrial staining

Synchronized eggs were incubated on nematode growth medium plates containing 500 nM MitoTracker Deep Red (Molecular Probe) for 2 days. The worms were washed by M9 and mounted on agarose pads. Pictures were taken by Leica confocal microscopy SP5 X. The deconvolution was conducted using Huygens Professional (Scientific Volume Imaging), to examine localization of MML-1::GFP and MXL-2::GFP to mitochondria.

### RNA sequencing

For each genotype, more than 300 day 1 adult worms were collected in Trizol in three independent biological replicates. One microgram of of RNA was extracted using miRNAeasy Mini Kit (QIAGEN). polyA+ mRNA was isolated from 500 ng total RNA with NEBNext Poly(A) mRNA Magnetics Isolation Module (New England Biolabs). RNA-seq libraries were prepared with the NEBNext Ultra Directional RNA Library Prep Kit for Illumina (New England Biolabs). Quality and quantity was assessed at all steps by capillary electrophoresis (Agilent Bioanalyser and Agilent Tapestation). Libraries were quantified by fluorometry, immobilized and processed onto a flow cell with a cBot (Illumina) followed by sequencing-by-synthesis with TruSeq v3 chemistry on a HiSeq2500 at the Max Planck Genome Center (Cologne, Germany). Reads were trimmed for adapter and barcodes using the Flexbar version 2.5 software^51^. Alignment of the reads was done using the Tophat version 2.0.13 software against the Wormbase genome (WBcel235_79). The Tuxedo/cufflinks version 2.2.1 software pipeline was used to perform differential gene expression analysis on pairwise comparisons of the different samples. Dispersion was calculated per condition (genotypes) and differential expressed genes (*q*-value<0.05) of each pairwise comparison were identified. GO annotation and enrichment analysis was performed using the DAVID bioinformatics resources database.

### Cell culture experiments

HeLa (P5-P10) cells (American Type Culture Collection, USA; ATCC-CCL-2) were maintained in DMEM medium (Gibco), supplemented with 10% fetal bovine serum (Gibco). Transfections of MondoA (Santa Cruz Biotechnology), ChREBP (Santa Cruz Biotechnology) and TFEB (GE Dharmacon) siRNA with respective control siRNAs were performed using INTERFERin (Polyplus Transfection) according to the manufacturer’s instruction. For TFEB nuclear localization assay, cells were starved in the starvation medium (140 mM NaCl, 1 mM CaCl_2_, 1 mM MgCl_2_, 5 mM glucose, 1% BSA and 20 mM HEPES, pH 7.4)^52^ for 4 h (for immunohistochemistry and western blotting) and fixed by 4% paraformaldehyde followed by MeOH fixation. Immunohistochemistry was performed using anti-TFEB antibody (1/500, rabbit or goat, Cell Signaling, #4240 or Abcam ab2636, respectively) followed by anti-rabbit or anti-goat secondary Alexa 488 antibody (1/500, donkey, ThermoFisher Scientific, A-11055) incubation. For each experimental condition, more than 100 cells were scored for their TFEB nuclear localization in a blinded manner and in at least 3 different biological replicates. For each biological replicate, the quantity of *MondoA* or *ChREBP* gene knockdown was measured by western blottings. For western blotting, cells were collected in RIPA buffer (Cell Signaling) with cOmplete protease inhibitor (Roche). The following antibodies and concentrations were used: anti-MondoA (1/1,000, rabbit, Santa Cruz Biotechnology, sc-133397), anti-ChREBP (1/1,000, goat, Santa Cruz Biotechnology, sc-21189), anti-TFEB (1/1,000, rabbit, Novus, NB100-1030) and anti-b-actin (1/10,000, mouse, Cell Signaling, #3700). For qRT–PCR experiments, cells were starved for 8 h in starvation medium and collected in Trizol (Roche). qPCR primers used for gene expression analyses are listed in Supplementary Table 3.

### Statistical analysis

Results are presented as mean±s.e.m. Statistical tests were preformed with either one-way analysis of variance with Tukey’s test or *t*-test using GraphPad Prism (GraphPad Software) or Excel (Microsoft Office 2011).

## Additional information

**Accession codes:** RNA-seq data have been deposited in the ArrayExpress database under accession code E-MTAB-3686.

**How to cite this article:** Nakamura, S. *et al*. Mondo complexes regulate TFEB via TOR inhibition to promote longevity in response to gonadal signals. *Nat. Commun.* 7:10944 doi: 10.1038/ncomms10944 (2016).

## Supplementary Material

Supplementary Figures and Supplementary Tables Supplementary Figures 1-9 and Supplementary Tables 1-4

Supplementary Data 1 GO Enrichment Analysis for Different Comparisons. Table of gene ontology (GO) term enrichment analysis for differential expressed gene (DEG) sets, given as sheet names. DEG sets were queried for GO categories BP_FAT, CC_FAT and MF_FAT at https://david.ncifcrf.gov for the enrichment analysis and merged with DEG tables (script 'aDiff' available at https://github.com/mpg-age-bioinformatics/htseq-tools/). Enrichment is stored in column 'Fold Enrichment', significance e.g. in 'FDR' and DEGs as list in 'log2.fold_change.', significance as list in 'q_value', for each 'Term' in each 'Category', respectively

Supplementary Data 2 KEGG Pathway Enrichment Analysis for Different Comparisons. Same as Supplementary Data 1, but queried for the category KEGG_PATHWAY.

## References

[b1] Kenyon C. J. The genetics of ageing. Nature 464, 504–512 (2010). 20336132 10.1038/nature08980

[b2] Kenyon C., Chang J., Gensch E., Rudner A. & Tabtiang R. A *C. elegans* mutant that lives twice as long as wild type. Nature 366, 461–464 (1993). 8247153 10.1038/366461a0

[b3] Tullet J. M. . Direct inhibition of the longevity-promoting factor SKN-1 by insulin-like signaling in *C. elegans*. Cell 132, 1025–1038 (2008). 18358814 10.1016/j.cell.2008.01.030 PMC2367249

[b4] Hsu A. L., Murphy C. T. & Kenyon C. Regulation of aging and age-related disease by DAF-16 and heat-shock factor. Science 300, 1142–1145 (2003). 12750521 10.1126/science.1083701

[b5] Panowski S. H., Wolff S., Aguilaniu H., Durieux J. & Dillin A. PHA-4/Foxa mediates diet-restriction-induced longevity of *C. elegans*. Nature 447, 550–555 (2007). 17476212 10.1038/nature05837

[b6] Bishop N. A. & Guarente L. Two neurons mediate diet-restriction-induced longevity in *C. elegans*. Nature 447, 545–549 (2007). 17538612 10.1038/nature05904

[b7] Heestand B. N. . Dietary restriction induced longevity is mediated by nuclear receptor NHR-62 in Caenorhabditis elegans. PLoS Genet. 9, e1003651 (2013). 23935515 10.1371/journal.pgen.1003651 PMC3723528

[b8] Thondamal M., Witting M., Schmitt-Kopplin P. & Aguilaniu H. Steroid hormone signalling links reproduction to lifespan in dietary-restricted *Caenorhabditis elegans*. Nat. Commun. 5, 4879 (2014). 25209682 10.1038/ncomms5879

[b9] Hsin H. & Kenyon C. Signals from the reproductive system regulate the lifespan of *C. elegans*. Nature 399, 362–366 (1999). 10360574 10.1038/20694

[b10] Gerisch B., Weitzel C., Kober-Eisermann C., Rottiers V. & Antebi A. A hormonal signaling pathway influencing *C. elegans* metabolism, reproductive development, and life span. Dev. Cell 1, 841–851 (2001). 11740945 10.1016/s1534-5807(01)00085-5

[b11] Gerisch B. . A bile acid-like steroid modulates *Caenorhabditis elegans* lifespan through nuclear receptor signaling. Proc. Natl Acad. Sci. USA 104, 5014–5019 (2007). 17360327 10.1073/pnas.0700847104 PMC1821127

[b12] Yamawaki T. M. . The somatic reproductive tissues of *C. elegans* promote longevity through steroid hormone signaling. PLoS Biol. 8, e1000468 (2010). 20824162 10.1371/journal.pbio.1000468 PMC2930862

[b13] Goudeau J. . Fatty acid desaturation links germ cell loss to longevity through NHR-80/HNF4 in C. elegans. PLoS Biol. 9, e1000599 (2011). 21423649 10.1371/journal.pbio.1000599 PMC3057950

[b14] Lapierre L. R., Gelino S., Melendez A. & Hansen M. Autophagy and lipid metabolism coordinately modulate life span in germline-less *C. elegans*. Curr. Biol. 21, 1507–1514 (2011). 21906946 10.1016/j.cub.2011.07.042 PMC3191188

[b15] Ratnappan R. . Germline signals deploy NHR-49 to modulate fatty-acid beta-oxidation and desaturation in somatic tissues of C. elegans. PLoS Genet. 10, e1004829 (2014). 25474470 10.1371/journal.pgen.1004829 PMC4256272

[b16] Melendez A. . Autophagy genes are essential for dauer development and life-span extension in *C. elegans*. Science 301, 1387–1391 (2003). 12958363 10.1126/science.1087782

[b17] Jia K. & Levine B. Autophagy is required for dietary restriction-mediated life span extension in *C. elegans*. Autophagy 3, 597–599 (2007). 17912023 10.4161/auto.4989

[b18] Hansen M. . A role for autophagy in the extension of lifespan by dietary restriction in C. elegans. PLoS Genet. 4, e24 (2008). 18282106 10.1371/journal.pgen.0040024 PMC2242811

[b19] Lapierre L. R. . The TFEB orthologue HLH-30 regulates autophagy and modulates longevity in *Caenorhabditis elegans*. Nat. Commun. 4, 2267 (2013). 23925298 10.1038/ncomms3267 PMC3866206

[b20] Pyo J. O. . Overexpression of Atg5 in mice activates autophagy and extends lifespan. Nat. Commun. 4, 2300 (2013). 23939249 10.1038/ncomms3300 PMC3753544

[b21] Settembre C. . TFEB links autophagy to lysosomal biogenesis. Science 332, 1429–1433 (2011). 21617040 10.1126/science.1204592 PMC3638014

[b22] Sardiello M. . A gene network regulating lysosomal biogenesis and function. Science 325, 473–477 (2009). 19556463 10.1126/science.1174447

[b23] Pickett C. L., Breen K. T. & Ayer D. E. A *C. elegans* Myc-like network cooperates with semaphorin and Wnt signaling pathways to control cell migration. Dev. Biol. 310, 226–239 (2007). 17826759 10.1016/j.ydbio.2007.07.034 PMC2077855

[b24] Filhoulaud G., Guilmeau S., Dentin R., Girard J. & Postic C. Novel insights into ChREBP regulation and function. Trends Endocrinol. Metab. 24, 257–268 (2013). 23597489 10.1016/j.tem.2013.01.003

[b25] Havula E. & Hietakangas V. Glucose sensing by ChREBP/MondoA-Mlx transcription factors. Semin. Cell Dev. Biol. 23, 640–647 (2012). 22406740 10.1016/j.semcdb.2012.02.007

[b26] Johnson D. W. . The *Caenorhabditis elegans* Myc-Mondo/Mad complexes integrate diverse longevity signals. PLoS Genet. 10, e1004278 (2014). 24699255 10.1371/journal.pgen.1004278 PMC3974684

[b27] Sans C. L., Satterwhite D. J., Stoltzman C. A., Breen K. T. & Ayer D. E. MondoA-Mlx heterodimers are candidate sensors of cellular energy status: mitochondrial localization and direct regulation of glycolysis. Mol. Cell. Biol. 26, 4863–4871 (2006). 16782875 10.1128/MCB.00657-05 PMC1489152

[b28] O'Rourke E. J. & Ruvkun G. MXL-3 and HLH-30 transcriptionally link lipolysis and autophagy to nutrient availability. Nat. Cell Biol. 15, 668–676 (2013). 23604316 10.1038/ncb2741 PMC3723461

[b29] Settembre C. . TFEB controls cellular lipid metabolism through a starvation-induced autoregulatory loop. Nat. Cell Biol. 15, 647–658 (2013). 23604321 10.1038/ncb2718 PMC3699877

[b30] Efeyan A., Comb W. C. & Sabatini D. M. Nutrient-sensing mechanisms and pathways. Nature 517, 302–310 (2015). 25592535 10.1038/nature14190 PMC4313349

[b31] Settembre C. . A lysosome-to-nucleus signalling mechanism senses and regulates the lysosome via mTOR and TFEB. EMBO J. 31, 1095–1108 (2012). 22343943 10.1038/emboj.2012.32 PMC3298007

[b32] Martina J. A., Chen Y., Gucek M. & Puertollano R. MTORC1 functions as a transcriptional regulator of autophagy by preventing nuclear transport of TFEB. Autophagy 8, 903–914 (2012). 22576015 10.4161/auto.19653 PMC3427256

[b33] Roczniak-Ferguson A. . The transcription factor TFEB links mTORC1 signaling to transcriptional control of lysosome homeostasis. Sci. Signal. 5, ra42 (2012). 22692423 10.1126/scisignal.2002790 PMC3437338

[b34] Bonfils G. . Leucyl-tRNA synthetase controls TORC1 via the EGO complex. Mol. Cell 46, 105–110 (2012). 22424774 10.1016/j.molcel.2012.02.009

[b35] Han J. M. . Leucyl-tRNA synthetase is an intracellular leucine sensor for the mTORC1-signaling pathway. Cell 149, 410–424 (2012). 22424946 10.1016/j.cell.2012.02.044

[b36] Meissner B., Boll M., Daniel H. & Baumeister R. Deletion of the intestinal peptide transporter affects insulin and TOR signaling in *Caenorhabditis elegans*. J. Biol. Chem. 279, 36739–36745 (2004). 15155758 10.1074/jbc.M403415200

[b37] Essers P. B. . A long noncoding RNA on the ribosome is required for lifespan extension. Cell Rep. 10, 339–345 (2015). 25600869 10.1016/j.celrep.2014.12.029

[b38] Hansen M., Hsu A. L., Dillin A. & Kenyon C. New genes tied to endocrine, metabolic, and dietary regulation of lifespan from a *Caenorhabditis elegans* genomic RNAi screen. PLoS Genet. 1, 119–128 (2005). 16103914 10.1371/journal.pgen.0010017 PMC1183531

[b39] Riesen M. . MDL-1, a growth- and tumor-suppressor, slows aging and prevents germline hyperplasia and hypertrophy in *C. elegans*. Aging 6, 98–117 (2014). 24531613 10.18632/aging.100638 PMC3969279

[b40] Grove C. A. . A multiparameter network reveals extensive divergence between *C. elegans* bHLH transcription factors. Cell 138, 314–327 (2009). 19632181 10.1016/j.cell.2009.04.058 PMC2774807

[b41] Hine C. & Mitchell J. R. Calorie restriction and methionine restriction in control of endogenous hydrogen sulfide production by the transsulfuration pathway. Exp. Gerontol. 68, 26–32 (2015). 25523462 10.1016/j.exger.2014.12.010 PMC4464900

[b42] Gerstein M. B. . Integrative analysis of the *Caenorhabditis elegans* genome by the modENCODE project. Science 330, 1775–1787 (2010). 21177976 10.1126/science.1196914 PMC3142569

[b43] O'Shea J. M. & Ayer D. E. Coordination of nutrient availability and utilization by MAX- and MLX-centered transcription networks. Cold Spring Harb. Perspect. Med. 3, a014258 (2013). 24003245 10.1101/cshperspect.a014258 PMC3753723

[b44] Berman J. R. & Kenyon C. Germ-cell loss extends *C. elegans* life span through regulation of DAF-16 by kri-1 and lipophilic-hormone signaling. Cell 124, 1055–1068 (2006). 16530050 10.1016/j.cell.2006.01.039

[b45] Shen Y., Wollam J., Magner D., Karalay O. & Antebi A. A steroid receptor-microRNA switch regulates life span in response to signals from the gonad. Science 338, 1472–1476 (2012). 23239738 10.1126/science.1228967 PMC3909774

[b46] Robida-Stubbs S. . TOR signaling and rapamycin influence longevity by regulating SKN-1/Nrf and DAF-16/FoxO. Cell Metab. 15, 713–724 (2012). 22560223 10.1016/j.cmet.2012.04.007 PMC3348514

[b47] Sheaffer K. L., Updike D. L. & Mango S. E. The target of rapamycin pathway antagonizes pha-4/FoxA to control development and aging. Curr. Biol. 18, 1355–1364 (2008). 18804378 10.1016/j.cub.2008.07.097 PMC2615410

[b48] Kaadige M. R., Yang J., Wilde B. R. & Ayer D. E. MondoA-Mlx transcriptional activity is limited by mTOR-MondoA interaction. Mol. Cell. Biol. 35, 101–110 (2015). 25332233 10.1128/MCB.00636-14 PMC4295369

[b49] Hofmann J. W. . Reduced expression of MYC increases longevity and enhances healthspan. Cell 160, 477–488 (2015). 25619689 10.1016/j.cell.2014.12.016 PMC4624921

[b50] Horn M. . DRE-1/FBXO11-dependent degradation of BLMP-1/BLIMP-1 governs *C. elegans* developmental timing and maturation. Dev. Cell 28, 697–710 (2014). 24613396 10.1016/j.devcel.2014.01.028 PMC4040421

[b51] Dodt M., Roehr J. T., Ahmed R. & Dieterich C. FLEXBAR-flexible barcode and adapter processing for next-generation sequencing platforms. Biology (Basel) 1, 895–905 (2012). 24832523 10.3390/biology1030895 PMC4009805

[b52] Axe E. L. . Autophagosome formation from membrane compartments enriched in phosphatidylinositol 3-phosphate and dynamically connected to the endoplasmic reticulum. J. Cell Biol. 182, 685–701 (2008). 18725538 10.1083/jcb.200803137 PMC2518708

