# Supplementary material for: Mondo complexes regulate TFEB via TOR inhibition to promote longevity in response to gonadal signals
Source: Nat Commun. 2016 Mar 22;7:10944. doi: 10.1038/ncomms10944 (PMC4804169; doi:10.1038/ncomms10944)
Supplement: Supplementary Figures and Supplementary Tables — Supplementary Figures 1-9 and Supplementary Tables 1-4 [file ncomms10944-s1.pdf]

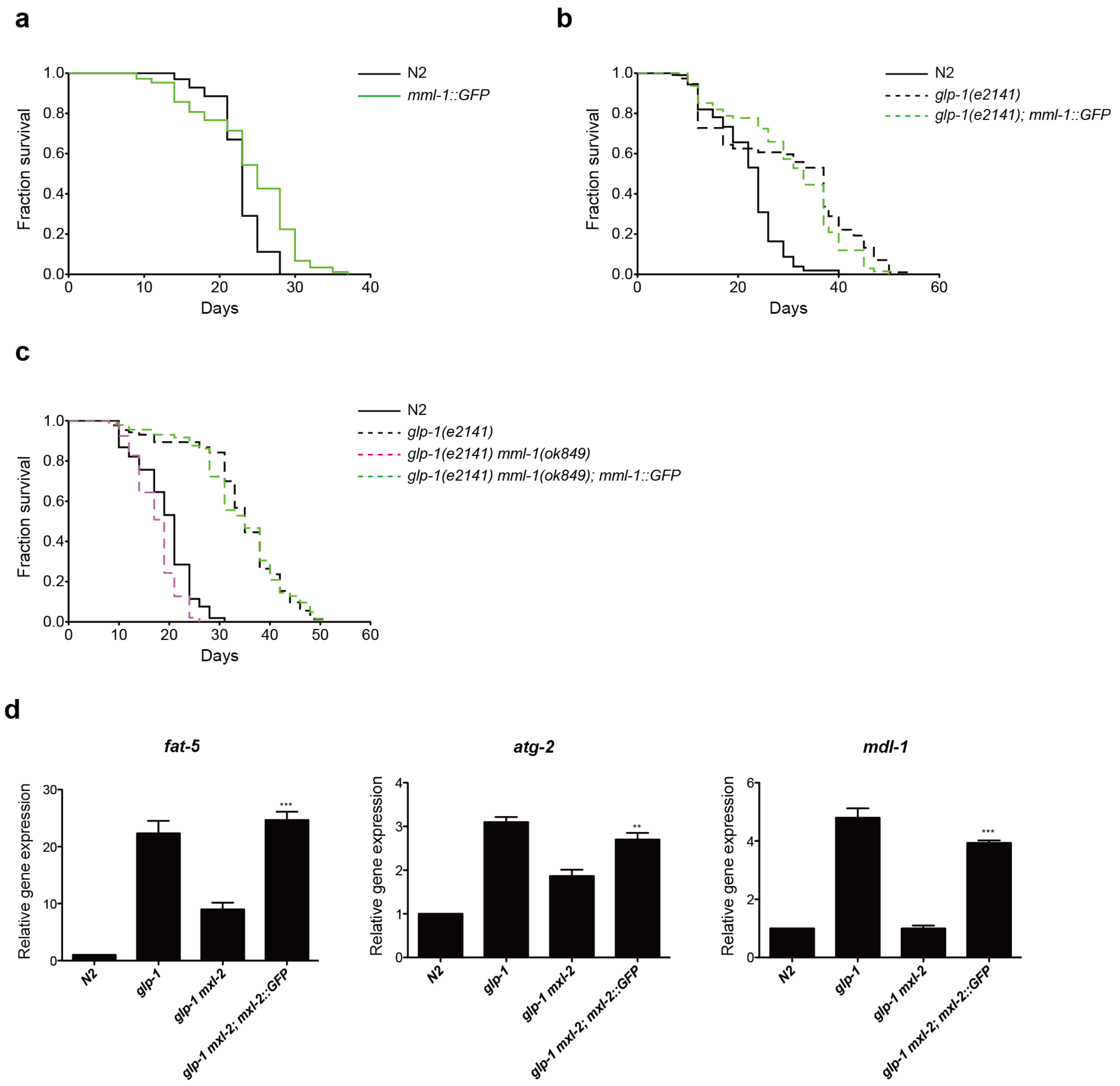

Supplementary Figure 1: MML-1 Overexpression Modestly Extends Wild Type Lifespan.

(a) *mml-1* overexpression modestly but significantly extends wild type lifespan (3/6 biological replicates). (b) *mml-1* overexpression does not further extend *glp-1* longevity. (c) MML-1::GFP rescues the short-lived phenotype of *glp-1mml-1*. See Supplementary Table 1 for details and repeats (a-c). (d) MXL-2::GFP rescues the expression of *mml-1/mxl-2* preferential target genes (See Fig.6c and Supplementary fig. 6d). The bars indicate mean ± s.e.m. from three biological replicates. P value relative to *glp-1mxl-2* (**P< 0.01, ***P< 0.001) was determined by *t*-test.

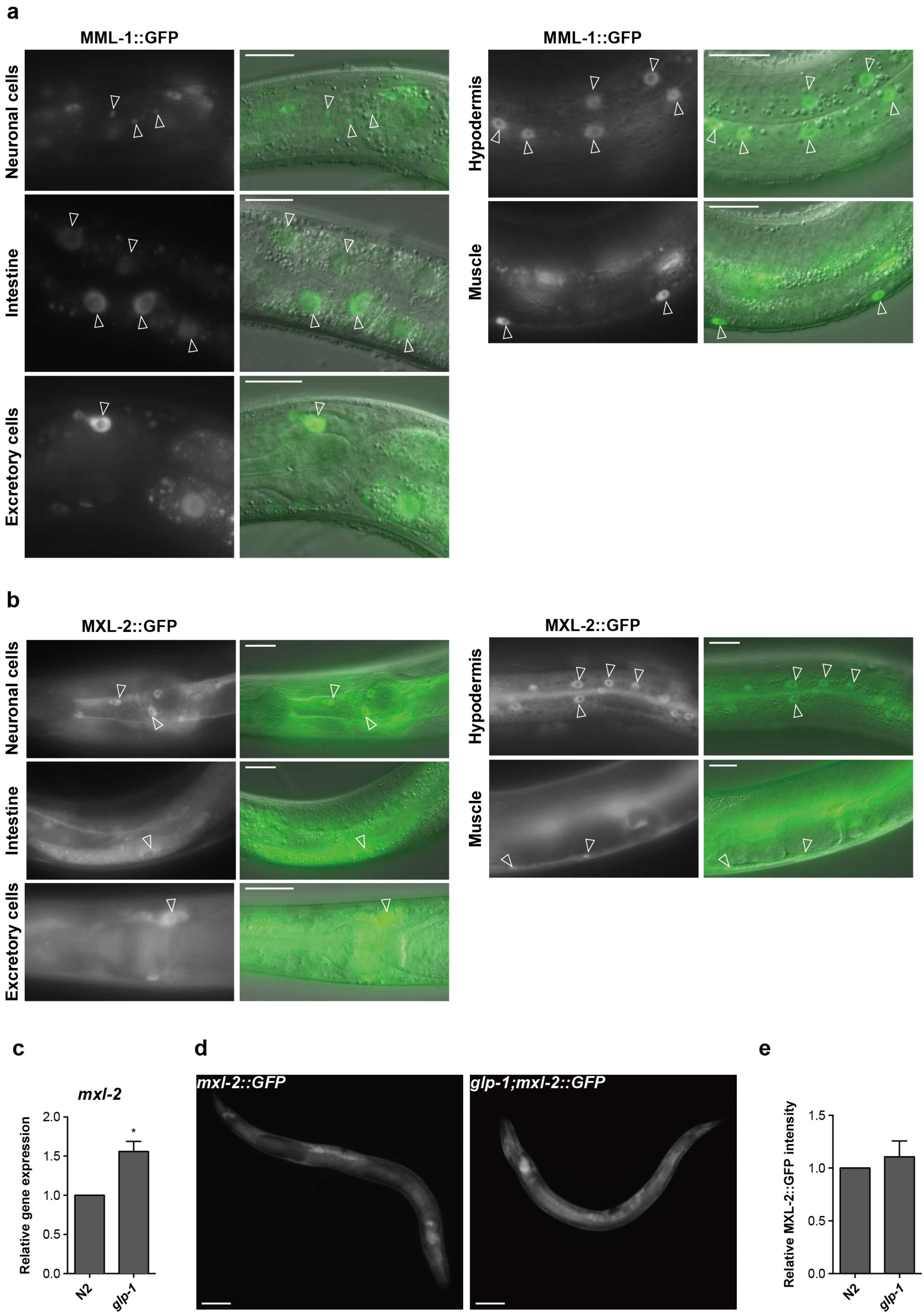

a
MML-1::GFP
Neuronal cells
Intestine
Excretory cells
MML-1::GFP
Hypodermis
Muscle
b
MXL-2::GFP
Neuronal cells
Intestine
Excretory cells
MXL-2::GFP
Hypodermis
Muscle
c
mxl-2
Relative gene expression
2.0
1.5
1.0
0.5
0.0
*
N2
glp-1
d
mxl-2::GFP
glp-1;mxl-2::GFP
e
Relative MXL-2::GFP intensity
1.5
1.0
0.5
0.0
N2
glp-1

Supplementary Figure 2: MML-1 and MXL-2 Expression Patterns.

(a) MML-1::GFP expression patterns at day1 adult stage. MML-1::GFP is found in the nucleus (empty arrowheads) of neuronal cells, intestine, excretory cells, hypodermis and muscle, but also found in the cytoplasmic region at higher magnification (Fig. 2a). (b) MXL-2::GFP expression pattern at day1 adult stage. MXL-2::GFP is smoothly distributed in neuronal cells, intestine, excretory cells, hypodermis and muscle (empty arrowheads). (c) qRT-PCR analysis of *mxl-2* transcripts. *mxl-2* is slightly upregulated in *glp-1* worms. The bars indicate mean ± s.e.m. from three biological replicates. P value (*P< 0.05) was determined by *t*-test. (d) The representative fluorescent images of MXL-2::GFP at day1 adult stage in N2 and *glp-1* background. (e) The quantification of MXL-2::GFP. The bars indicate mean ± s.e.m. from three biological replicates (>20 worms each). P value (P> 0.05) was determined by *t*-test. Scale bars, 20μm (a and b); 50μm (d).

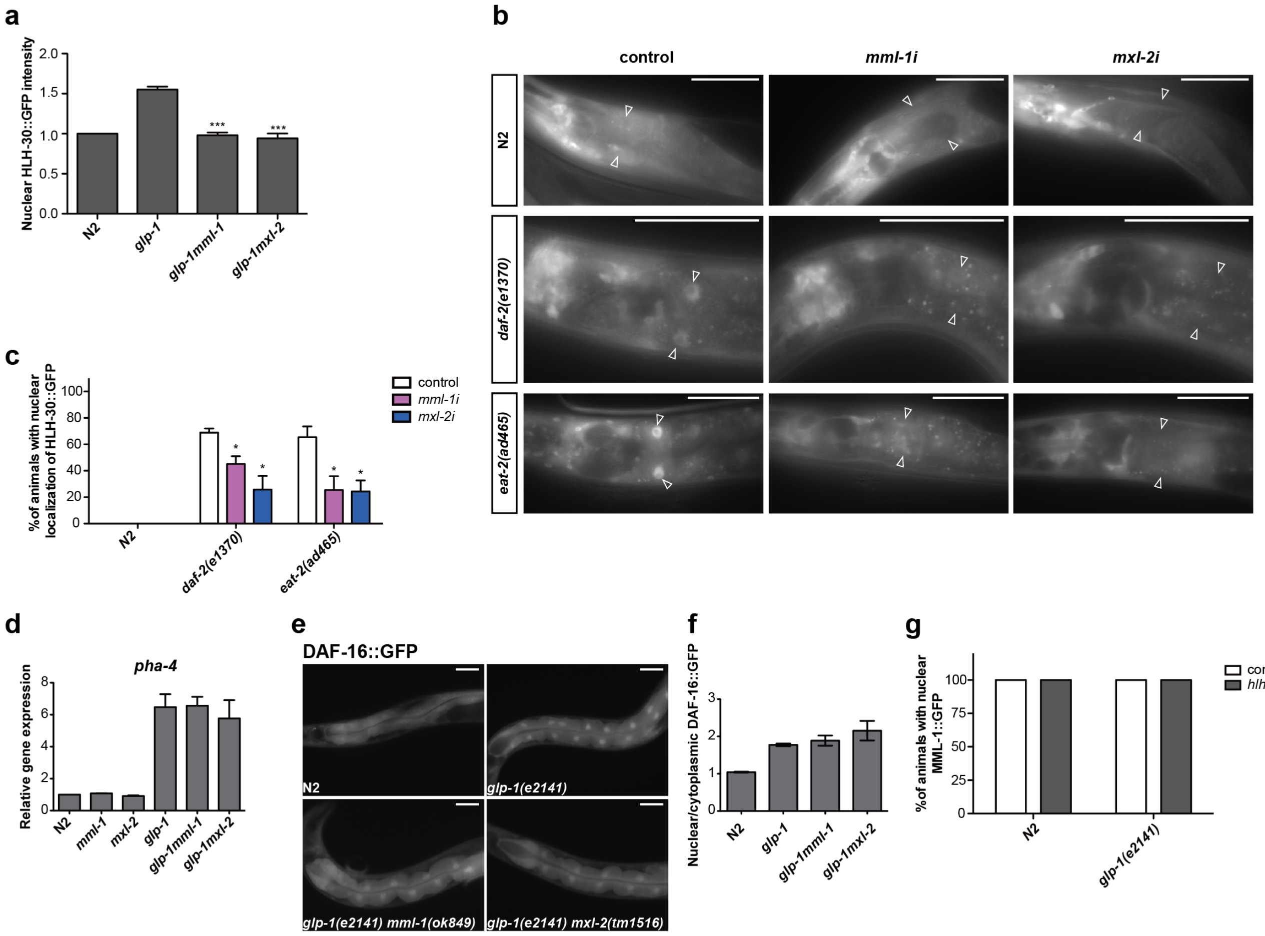

Supplementary Figure 3: MML-1/MXL-2 affects HLH-30 nuclear localization.

(a) The quantification of HLH-30::GFP intensity in the nucleus of day1 adult worms. The bars indicate mean ± s.e.m. from three biological replicates. P value (***P< 0.001) was determined by *t*-test relative to *glp-1*. (b) The representative pictures of HLH-30::GFP in day 1 adult stage wild type (N2), *daf-2 (e1370)* and *eat-2 (ad465)* background upon control, *mml-1* and *mxl-2* RNAi. HLH-30::GFP is localized in the anterior intestine in *daf-2* and *eat-2*, but these nuclear localization are impaired upon *mml-1* or *mxl-2* knockdown (arrowheads). We noticed that HLH-30::GFP levels are low in *daf-2* and *eat-2* background compared to wild type, therefore we took these pictures at different exposure time (N2, 100ms; *daf-2*, 500ms; *eat-2*, 300ms) (c) The quantification of HLH-30::GFP nuclear localization. The bars indicate mean ± s.e.m. from three biological replicates. P value (*P< 0.05) was determined by *t*-test. (d) qRT-PCR analysis of *pha-4* transcripts in indicated genotypes. The bars indicate mean ± s.e.m. from three biological replicates relative to N2. *pha-4* levels are not changed in *glp-1mml-1* and *glp-1mxl-2* compared to *glp-1*. P value (* P> 0.05) was determined by one-way ANOVA with Tukey's test relative to *glp-1*. (e) The representative fluorescent pictures of DAF-16::GFP in the intestine at day1 adult. DAF-16 nuclear localization is maintained in *glp-1mml-1* and *glp-1mxl-2*. (f) The quantification of DAF-16 nuclear localization. The ratios of fluorescence intensity in the nucleus and the cytoplasm are shown. The bars indicate mean ± s.e.m. from three biological replicates (>20 worms each). P value (P> 0.05) was determined

by *t*-test relative to *glp-1*. (g) The quantification of numbers of day 1 adult stage worms containing nuclear MML-1::GFP in N2 and *glp-1* strains upon control and *hlh-30* RNAi knockdown. In wild type background, MML-1::GFP is also present in the nucleus, albeit weakly. Scale bars, 50μm (b and e).

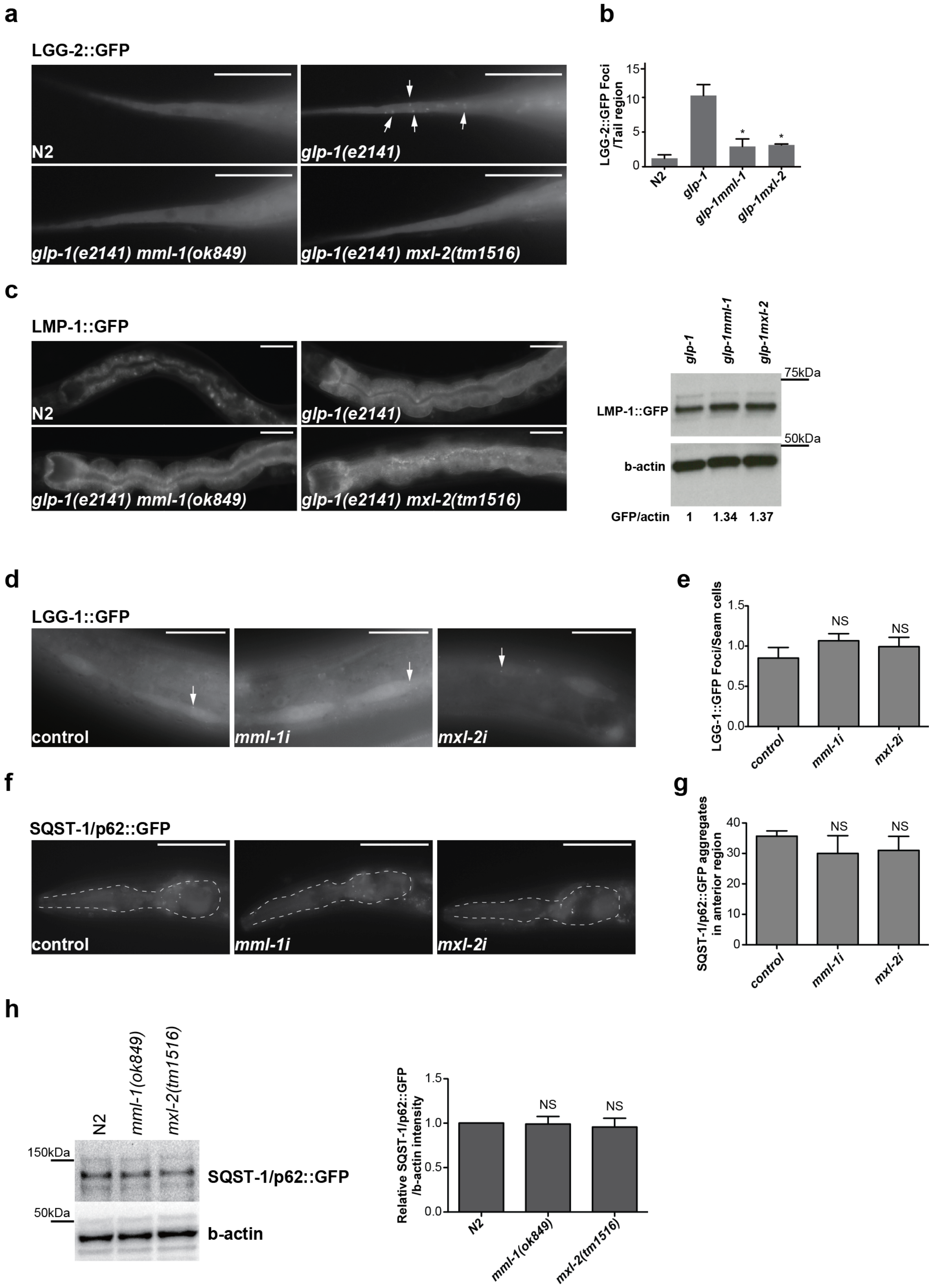

Supplementary Figure 4: MML-1/MXL-2 does not affects autophagy in wild type.

(a) The representative pictures showing LGG-2::GFP puncta (arrows) in indicated genotypes at L4 stage. (b) The quantification of LGG-2::GFP puncta in the tail region. The bars indicate mean ± s.e.m. from three biological replicates (>20 worms each). P value (*P< 0.05) was determined by *t*-test. (c) Representative fluorescent images of LMP-1::GFP of day1 adult worms (left). The LMP-

1::GFP was quantified by Western Blot (right). GFP bands were normalized to housekeeping gene b-actin bands. LMP-1::GFP levels were slightly upregulated in *glp-1mml-1* vs *glp-1mxl-2*. The results were reproduced in three independent experiments. (d) Representative LGG-1::GFP images at L4 stage upon control, *mml-1* and *mxl-2* RNAi. (e) Quantification of LGG-1::GFP puncta in seam cells. The bar represents mean ± s.e.m. from three biological replicates (>20 worms each) (*t*-test, NS [Not Significant]). *mml-1/mxl-2* knockdown by RNAi did not affect the numbers of LGG-1 foci. (f) Representative SQST-1/p62::GFP images at L4 stage upon control, *mml-1* and *mxl-2* RNAi (g) Quantification of SQST-1/p62::GFP aggregates in the pharyngeal region. The bar represents mean ± s.e.m. from three experiments (>20 worms each) (*t*-test, NS). (h) Representative Western Blot showing SQST-1::GFP and b-actin (left) and the quantification of blot (right) (*t*-test, NS). The results were reproduced in three independent experiments. Scale bars, 50μm (a and f); 20μm (c and d).

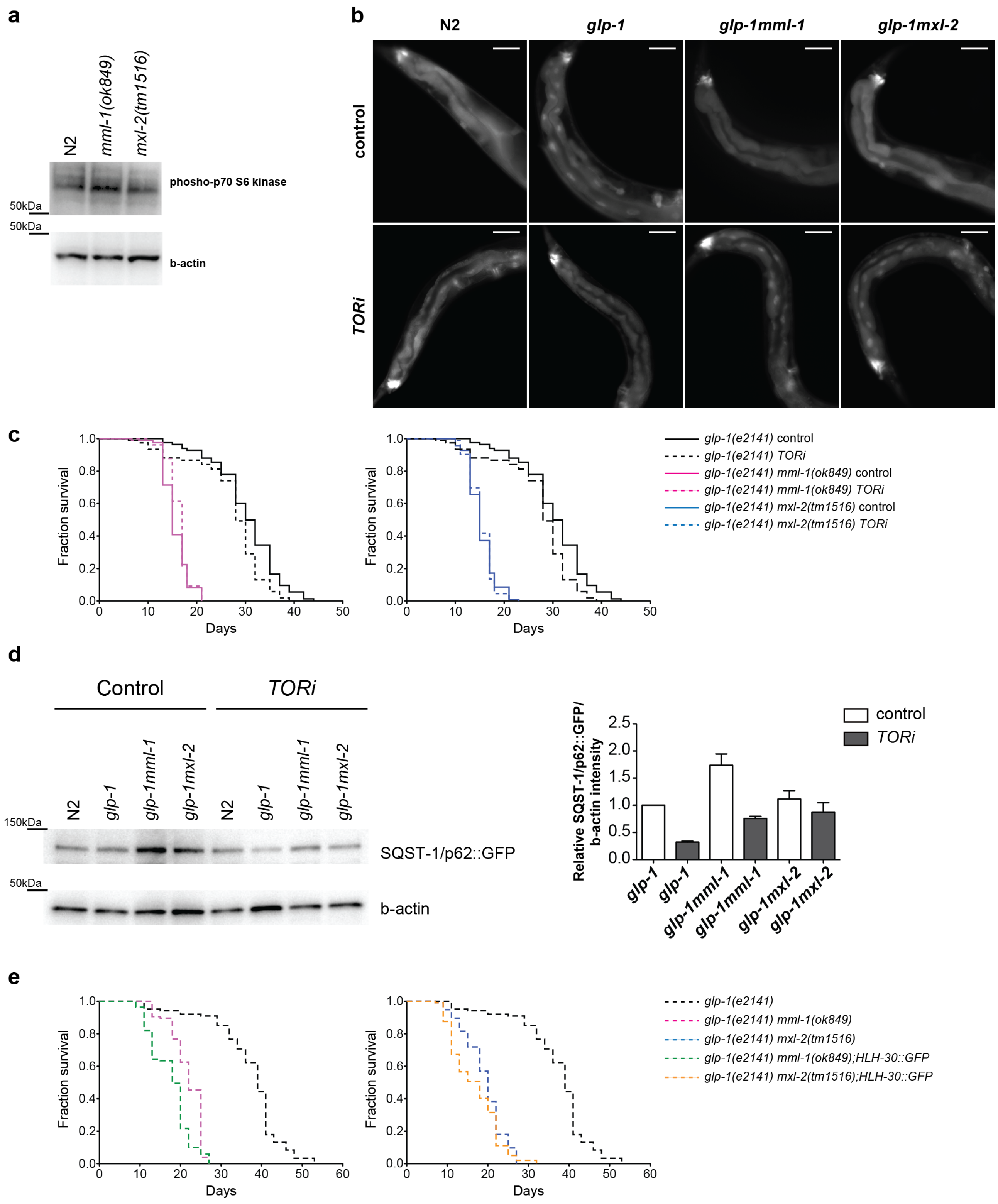

Supplementary Figure 5: HLH-30 Nuclear Localization is Not Sufficient to Rescue the Short-Lived Phenotype of *glp-1mml-1* and *glp-1mxl-2*.

(a) A representative Western Blot showing phosphorylation of p70 S6 kinase and b-actin. The phosphorylation of p70 S6 kinase did not change in *mml-1* and *mxl-2* mutants. The results were reproduced in three independent experiments. (b) Representative fluorescent pictures of HLH-30::GFP at day1 adult stage after treatment with control or TOR RNAi clones. TOR knockdown was carried out from egg onwards. TOR knockdown restores HLH-30 nuclear localization in *glp-*

1mml-1 and *glp-1mxl-2*. (c) TOR knockdown does not rescue the short-lived phenotype of *glp-1mml-1* and *glp-1mxl-2*. (d) A representative Western Blot showing SQST-1/p62::GFP and b-actin in indicated genotypes and conditions (left). The quantification of Western Blot analysis (right). The bars indicate mean ± s.e.m. from three biological replicates relative to *glp-1*. GFP intensity was normalized to the loading control, b-actin. TOR knockdown does not fully restore autophagy in *glp-1mml-1* and *glp-1mxl-2*. (e) HLH-30 overexpression does not rescue the shortevity of *glp-1mml-1* and *glp-1mxl-2*. See Supplementary Table 1 for details and repeats (c, e). Scale bars, 50μm (b).

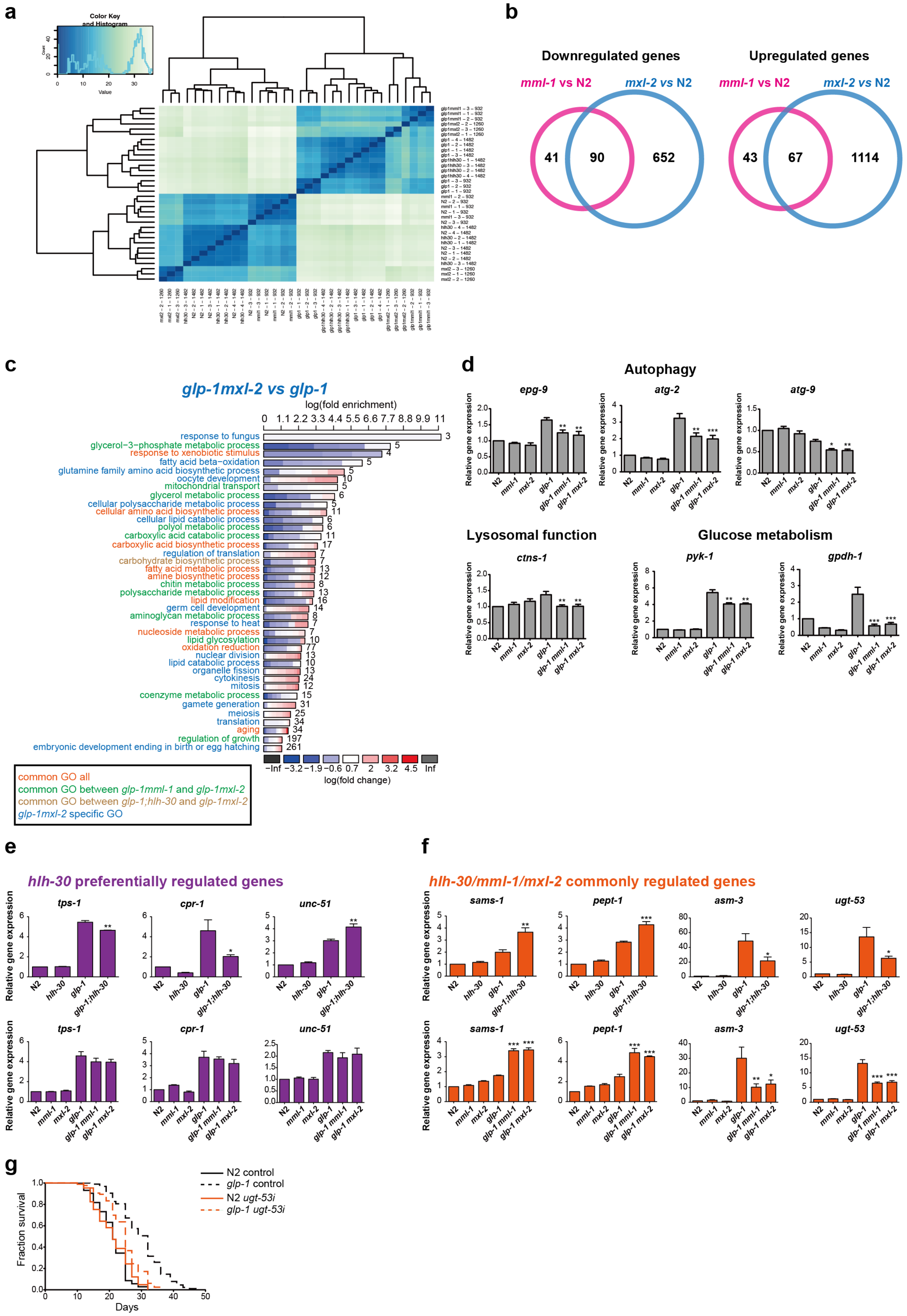
a
Color Key and Histogram
Count
Value
b
Downregulated genes
mml-1 vs N2
mxl-2 vs N2
41
90
652
Upregulated genes
mml-1 vs N2
mxl-2 vs N2
43
67
1114
c
glp-1mxl-2 vs glp-1
log(fold enrichment)
response to fungus
glycerol−3−phosphate metabolic process
response to xenobiotic stimulus
fatty acid beta−oxidation
glutamine family amino acid biosynthetic process
oocyte development
mitochondrial transport
glycerol metabolic process
cellular polysaccharide metabolic process
cellular amino acid biosynthetic process
cellular lipid catabolic process
polyol metabolic process
carboxylic acid catabolic process
carboxylic acid biosynthetic process
regulation of translation
carbohydrate biosynthetic process
fatty acid metabolic process
amine biosynthetic process
chitin metabolic process
polysaccharide metabolic process
lipid modification
germ cell development
aminoglycan metabolic process
response to heat
nucleoside metabolic process
lipid glycosylation
oxidation reduction
nuclear division
lipid catabolic process
organelle fission
cytokinesis
mitosis
coenzyme metabolic process
gamete generation
meiosis
translation
aging
regulation of growth
embryonic development ending in birth or egg hatching
log(fold change)
common GO all
common GO between glp-1mml-1 and glp-1mxl-2
common GO between glp-1;hlh-30 and glp-1mxl-2
glp-1mxl-2 specific GO
d
Autophagy
epg-9
atg-2
atg-9
Lysosomal function
ctns-1
Glucose metabolism
pyk-1
gpdh-1
Relative gene expression
N2
mml-1
mxl-2
glp-1
glp-1 mml-1
glp-1 mxl-2
e
hlh-30 preferentially regulated genes
tps-1
cpr-1
unc-51
hlh-30
glp-1;hlh-30
f
hlh-30/mml-1/mxl-2 commonly regulated genes
sams-1
pept-1
asm-3
ugt-53
g
N2 control
glp-1 control
N2 ugt-53i
glp-1 ugt-53i
Fraction survival
Days

Supplementary Figure 6: **Transcriptome Analysis of *hlh-30* and *mml-1/mxl-2* Reveals Distinct and Overlapping Target Genes.** (a) Hierarchical clustering analysis of RNA-seq samples based on the log fold changes of expression counts. (b) The Venn diagram shows the comparison of DEG between *mml-1* vs N2 and *mxl-2* vs N2. (c) GO enrichment analysis for DEG of *glp-1mxl-2* vs *glp-1*. (d) qRT-PCR analysis of autophagy genes (*epg-9*, *atg-2* and *atg-9*), lysosomal genes (*ctns-1*) and glucose metabolism genes (*pyk-1* and *gpdh-1*) The bars represent mean ± s.e.m. from three biological replicates. (e and f) qRT-PCR analysis of *hlh-30* preferentially regulated genes such as *tps-1, cpr-1* and *unc-51* and *hlh-30/mml-1/mxl-2* commonly regulated genes including *sams-1, pept-1, asm-3* and *ugt-53.* The bars represent mean ± s.e.m. from three biological replicates. (g) *hlh-30* and *mml-1/mxl-2* commonly regulated genes, *ugt-53* knockdown abolish *glp-1* longevity but do not affect N2 lifespan. See Supplementary Table 1 for details and repeats. (d-f) P value (* $P< 0.05$, ** $P< 0.01$ and *** $P< 0.001$) was determined by one-way ANOVA with Tukey's test relative to *glp-1*.

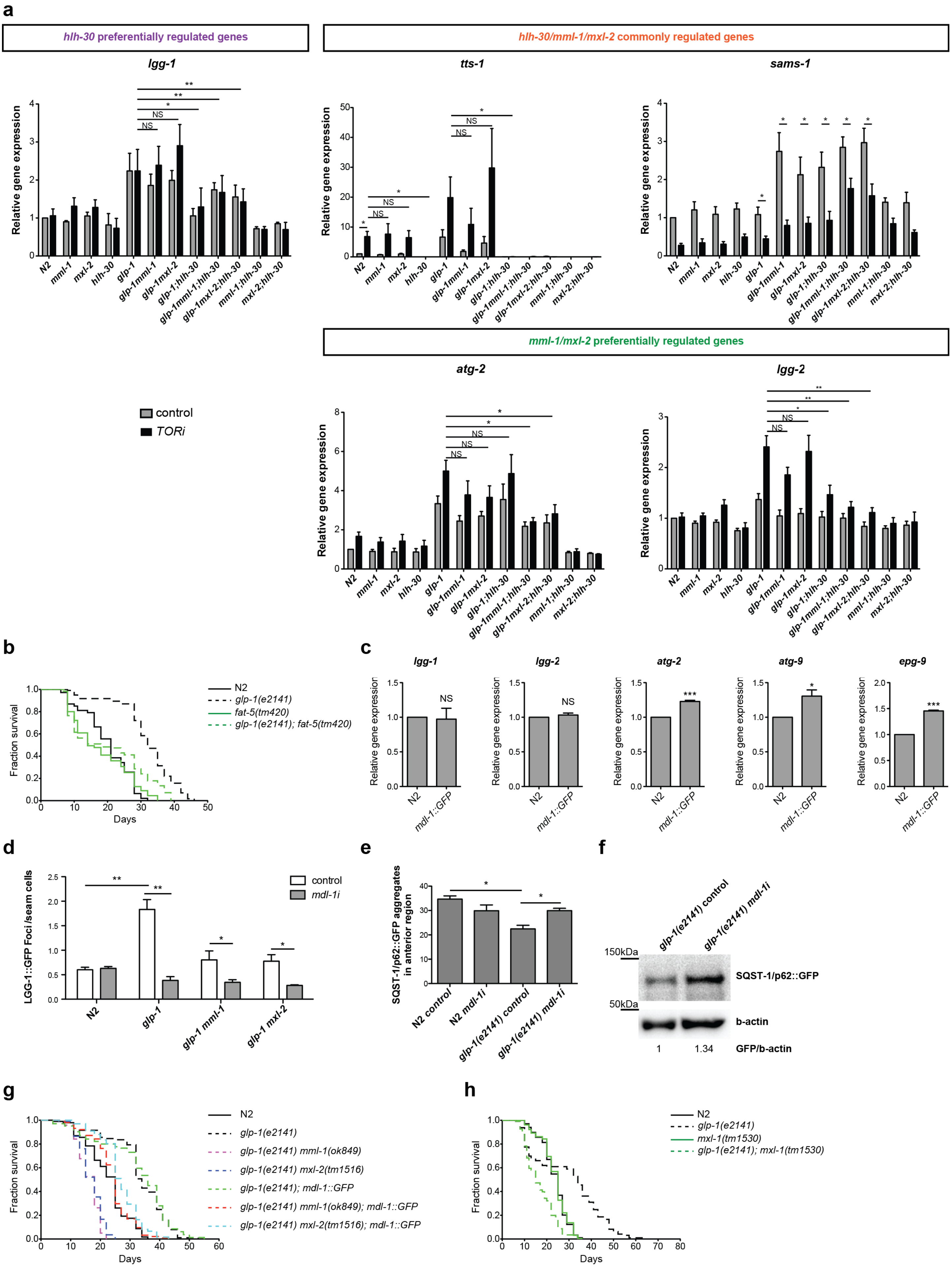
a
hlh-30 preferentially regulated genes
hlh-30/mml-1/mxl-2 commonly regulated genes
lgg-1
tts-1
sams-1
Relative gene expression
mml-1/mxl-2 preferentially regulated genes
atg-2
lgg-2
control
TORi
N2
mml-1
mxl-2
hlh-30
glp-1
glp-1mml-1
glp-1mxl-2
glp-1;hlh-30
glp-1mml-1;hlh-30
glp-1mxl-2;hlh-30
mml-1;hlh-30
mxl-2;hlh-30
b
N2
glp-1(e2141)
fat-5(tm420)
glp-1(e2141); fat-5(tm420)
Fraction survival
Days
c
lgg-1
lgg-2
atg-2
atg-9
epg-9
mdl-1::GFP
d
LGG-1::GFP Foci /seam cells
control
mdl-1i
glp-1 mml-1
glp-1 mxl-2
e
SQST-1/p62::GFP aggregates in anterior region
N2 control
N2 mdl-1i
glp-1(e2141) control
glp-1(e2141) mdl-1i
f
150kDa
50kDa
SQST-1/p62::GFP
b-actin
1
1.34
GFP/b-actin
g
glp-1(e2141) mml-1(ok849)
glp-1(e2141) mxl-2(tm1516)
glp-1(e2141); mdl-1::GFP
glp-1(e2141) mml-1(ok849); mdl-1::GFP
glp-1(e2141) mxl-2(tm1516); mdl-1::GFP
h
mxl-1(tm1530)
glp-1(e2141); mxl-1(tm1530)

Supplementary Figure 7: HLH transcription factors mediate TOR signaling outputs to various extents. (a) qRT-PCR analysis of *lgg-1*, *tts-1*, *sams-1*, *atg-2* and *lgg-2* after treatment with control or TOR RNAi in indicated genotypes. The bars indicate mean ± s.e.m. relative to N2 fed with control RNAi from three biological replicates. P value (* P< 0.05 and ** P< 0.01) was determined by one-way ANOVA with Tukey's test. (b) *fat-5 (tm420)* deletion suppresses *glp-1* longevity. (c) qRT-PCR analysis of autophagy genes in N2 and *mdl-1* overexpression strains. *mdl-1* overexpression induces the expression of some of the autophagy genes. The bar represents mean ± s.e.m. from three experiments. P value (*P< 0.05, ***P<0.001) was determined by *t*-test. (d) Quantification of LGG-1::GFP puncta in seam cells. The bar represents mean ± s.e.m. from three biological replicates (>20 worms each). P value (*P< 0.05, **P< 0.01) was determined by *t*-test. *mdl-1* RNAi decreases the numbers of LGG-1 foci in *glp-1* background. In addition, *mdl-1* RNAi further decreases the numbers of LGG-1 foci in *glp-1mml-1* and *glp-1mxl-2*. (e) Quantification of SQST-1/p62::GFP foci in the pharyngeal region. The bar represents mean ± s.e.m. from three experiments (>20 worms each). P value (*P< 0.05) relative to N2 control or *glp-1* control was determined by *t*-test. *mdl-1* RNAi knockdown increases SQST-1/p62::GFP foci. (f) Representative Western Blot showing SQST-1::GFP and loading control, b-actin. *mdl-1* RNAi knockdown increases the relative SQST-1/p62::GFP level normalized to b-actin in *glp-1* background in 3 different biological repeats. (g) MDL-1::GFP transgene partially rescues the shortevity of *glp-1mml-1* and *glp-1mxl-2*. (h) *mxl-1* is required for *glp-1* longevity. See Supplementary Table 1 for details and repeats (b, g and h).

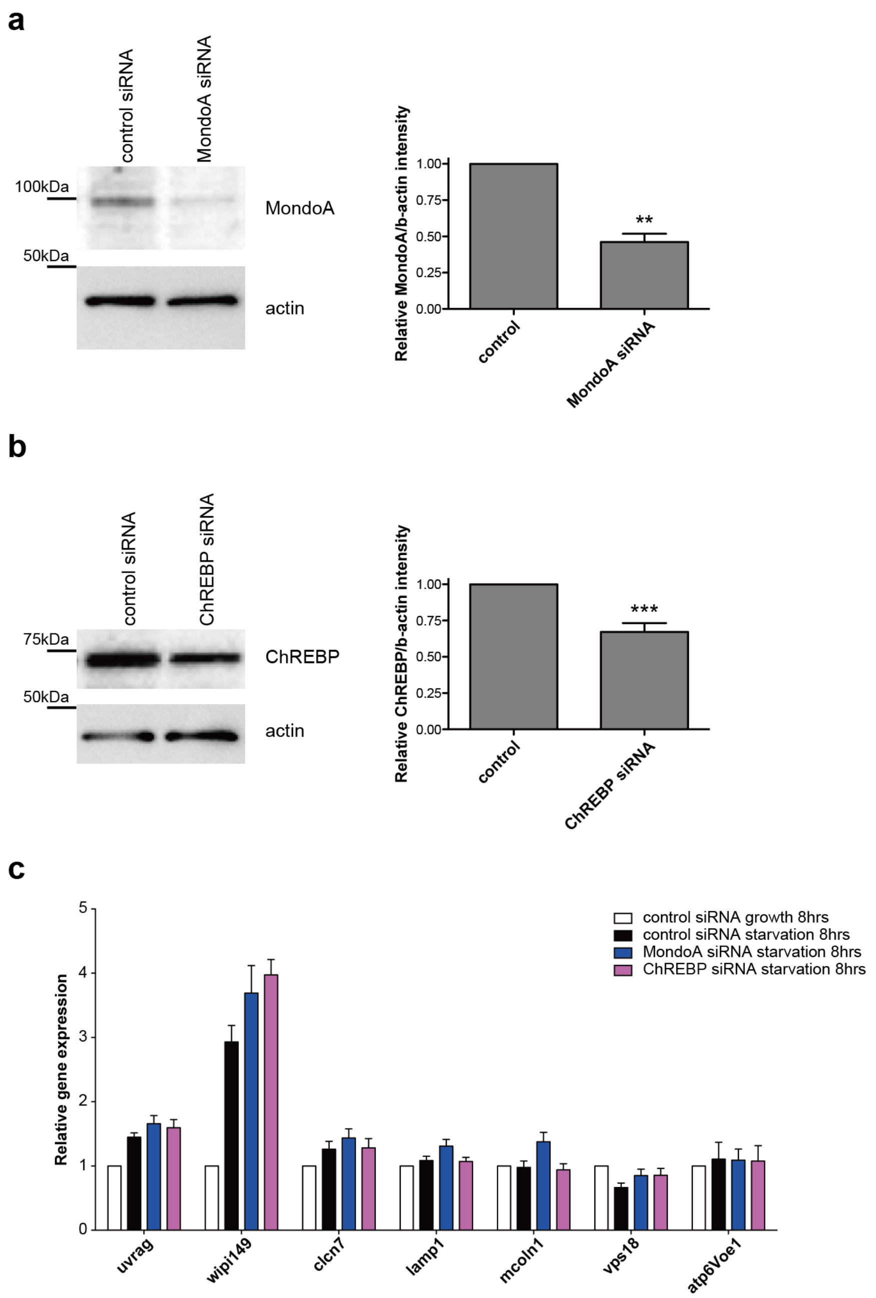

Supplementary Figure 8: MondoA and ChREBP Knockdown Affect a Subset of TFEB Target Genes.
(a and b) A representative Western Blot and bar graphs showing that MondoA or ChREBP siRNA diminish MondoA (a) or ChREBP (b) protein levels, respectively. The bars indicate mean ± s.e.m. relative to control from three biological replicates. P value (** P< 0.01 and *** P< 0.001) was determined by *t*-test. (c) qRT-PCR analysis of TFEB target genes. These genes are not significantly changed upon MondoA or ChREBP siRNA compared to control siRNA starvation condition. The bar represents mean ± s.e.m. from 5 experiments relative to control siRNA 8 hours growth condition.

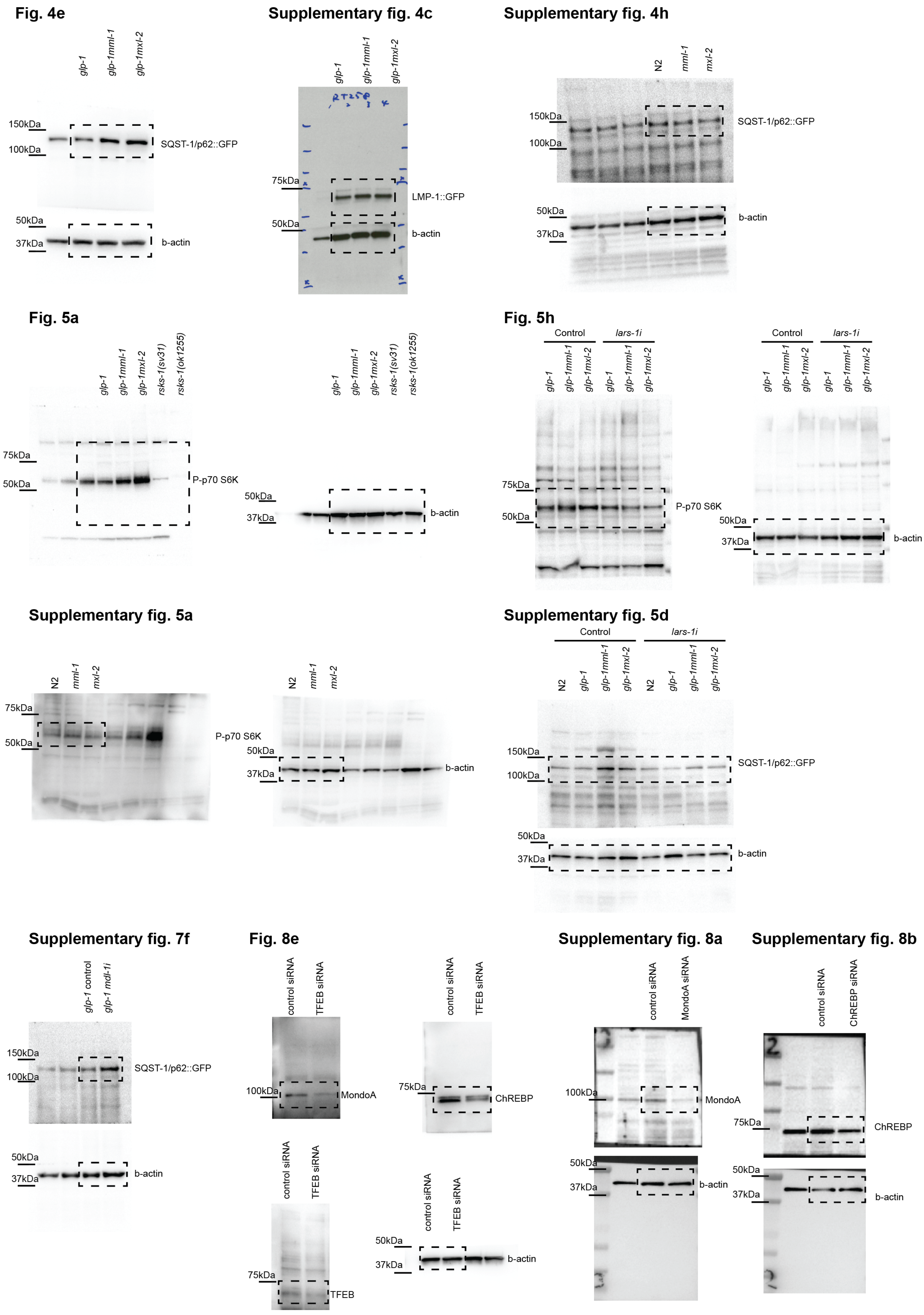

Supplementary Figure 9: Un-cropped Western Blot with size markers

Supplementary Table 1: Lifespan data.

Strains	Treatment /RNAi	Median LS	Median diff. from cntl.	* Animals	Max LS	Max diff. from cntl.	P value	Ref. Cntl.

***glp-1* lifespan**

Both *mml-1* and *mxl-2* are required for *glp-1* longevity.

Strains	Treatment /RNAi	Median LS	Median diff. from cntl.	* Animals	Max LS	Max diff. from cntl.	P value	Ref. Cntl.
N2		29		65/120	36			
mml-1 (ok849)		26	-10.34%	70/120	36	0.00%	0.0109	vs. N2
mxl-2 (tm1516)		17	-41.38%	88/120	31	-13.89%	<0.0001	vs. N2
glp-1 (e2141)		43	48.28	101/120	58	61.11%	<0.0001	vs. N2
glp-1 (e2141) mml-1 (ok849)		15	-65.12%	99/120	24	-58.62%	<0.0001	vs. *glp-1 (e2141)*
glp-1 (e2141) mxl-2 (tm1516)		12	-72.09%	101/120	26	-55.17%	<0.0001	vs. *glp-1 (e2141)*

Strains	Treatment /RNAi	Median LS	Median diff. from cntl.	* Animals	Max LS	Max diff. from cntl.	P value	Ref. Cntl.
N2		29		74/122	41			
mml-1 (ok849)		25	-13.79%	75/120	39	-13.79%	0.0073	vs. N2
mxl-2 (tm1516)		18	-37.93%	107120	32	-37.93%	<0.0001	vs. N2
glp-1 (e2141)		39	34.48%	87/120	52	34.48%	<0.0001	vs. N2
glp-1 (e2141) mml-1 (ok849)		14	-64.10%	100/120	27	-48.08%	<0.0001	vs. *glp-1 (e2141)*
glp-1 (e2141) mxl-2 (tm1516)		15	-61.54%	89/121	39	-25.00%	<0.0001	vs. *glp-1 (e2141)*

Strains	Treatment /RNAi	Median LS	Median diff. from cntl.	* Animals	Max LS	Max diff. from cntl.	P value	Ref. Cntl.
N2		26		81/120	38			
mml-1 (ok849)		24	-7.69%	75/120	38	0.00%	0.0048	vs. N2
mxl-2 (tm1516)		19	-26.92%	101/120	40	5.26%	<0.0001	vs. N2
glp-1 (e2141)		36	38.46%	96/120	56	47.37%	<0.0001	vs. N2
glp-1 (e2141) mml-1 (ok849)		17	-52.78%	105/120	33	-41.07%	<0.0001	vs. *glp-1 (e2141)*
glp-1 (e2141) mxl-2 (tm1516)		15	-58.33%	101/120	26	-53.57%	<0.0001	vs. *glp-1 (e2141)*

Germline ablation lifespan

Both *mml-1* and *mxl-2* are required for longevity conferred by Z1/Z2 ablation.

Strains	Treatment /RNAi	Median LS	Median diff. from cntl.	* Animals	Max LS	Max diff. from cntl.	P value	Ref. Cntl.
N2	mock	22		84/135	32			
mml-1 (ok849)	mock	22	0.00%	114/135	39	21.88%	NS	vs. N2 (mock)
mxl-2 (tm1516)	mock	15	-31.82%	104/135	34	6.25%	0.0001	vs. N2 (mock)
N2	Z1/Z2 ablated	39	77.27%	51/88	67	109.38%	<0.0001	vs. N2 (mock)
mml-1 (ok849)	Z1/Z2 ablated	18	-53.85%	22/74	25	-62.69%	<0.0001	vs. N2 (Z1/Z2 ablated)
mxl-2 (tm1516)	Z1/Z2 ablated	18	-53.85%	69/115	25	-62.69%	<0.0001	vs. N2 (Z1/Z2 ablated)

Strains	Treatment /RNAi	Median LS	Median diff. from cntl.	* Animals	Max LS	Max diff. from cntl.	P value	Ref. Cntl.
N2	mock	25		85/120	35			
mml-1 (ok849)	mock	25	0.00%	104/120	35	0.00%	0.0031	vs. N2 (mock)
mxl-2 (tm1516)	mock	20	-20.00%	91/120	35	0.00%	0.0533	vs. N2 (mock)
N2	Z1/Z2 ablated	35	40.00%	50/64	59	68.57%	<0.0001	vs. N2 (mock)
mml-1 (ok849)	Z1/Z2 ablated	22	-37.14%	27/68	27	-54.24%	<0.0001	vs. N2 (Z1/Z2 ablated)
mxl-2 (tm1516)	Z1/Z2 ablated	14	-60.00%	76/92	22	-62.71%	<0.0001	vs. N2 (Z1/Z2 ablated)

N2	mock	22		64/120	33			
mml-1 (ok849)	mock	19	-13.64%	90/120	30	-9.09%	0.0249	vs. N2 (mock)
mxl-2 (tm1516)	mock	16	-27.27%	74/120	33	0.00%	0.0029	vs. N2 (mock)
N2	Z1/Z2 ablated	38	72.73%	71/100	61	84.85%	<0.0001	vs. N2 (mock)
mml-1 (ok849)	Z1/Z2 ablated	16	-57.89%	65/89	28	-54.10%	<0.0001	vs. N2 (Z1/Z2 ablated)
mxl-2 (tm1516)	Z1/Z2 ablated	14	-63.16%	94/99	24	-60.66%	<0.0001	vs. N2 (Z1/Z2 ablated)

***daf-2* and *cco-1* RNAi lifespan**

Both *mml-1* and *mxl-2* are required for *daf-2* longevity.

mml-1 is not required for *cco-1* lifespan.

N2	L4440::Luc	25		62/120	35			
mml-1 (ok849)	L4440::Luc	18	-28.00%	98/120	29	-17.14%	<0.0001	vs. N2 (L4440:: Luc)
mxl-2 (tm1516)	L4440::Luc	18	-28.00%	105/123	29	-17.14%	<0.0001	vs. N2 (L4440:: Luc)
N2	*daf-2*	54	116.00%	73/120	71	102.86%	<0.0001	vs. N2 (L4440:: Luc)
mml-1 (ok849)	*daf-2*	25	-50.70%	83/120	35	-50.70%	<0.0001	vs. N2 (daf-2)
mxl-2 (tm1516)	*daf-2*	29	-45.07%	103/120	39	-45.07%	<0.0001	vs. N2 (daf-2)
N2	*cco-1*	32	28.00%	88/120	50	42.86%	<0.0001	vs. N2 (L4440:: Luc)
mml-1 (ok849)	*cco-1*	32	0.00%	80/120	53	6.00%	NS	vs. N2 (cco-1)
mxl-2 (tm1516)	*cco-1*	35	9.38%	74/120	64	28.00%	0.0029	vs. N2 (cco-1)

N2	L4440::Luc	22		66/120	36			
mml-1 (ok849)	L4440::Luc	17	-22.73%	101/120	29	-19.44%	<0.0001	vs. N2 (L4440:: Luc)
mxl-2 (tm1516)	L4440::Luc	17	-22.73%	99/120	26	-27.78%	<0.0001	vs. N2 (L4440:: Luc)
N2	*daf-2*	51	131.82%	45/119	66	83.33%	<0.0001	vs. N2 (L4440:: Luc)
mml-1 (ok849)	*daf-2*	24	-52.94%	67/120	39	-40.91%	<0.0001	vs. N2 (daf-2)
mxl-2 (tm1516)	*daf-2*	26	-49.02%	85/120	39	-40.91%	<0.0001	vs. N2 (daf-2)
N2	*cco-1*	29	31.82%	83/125	53	47.22%	<0.0001	vs. N2 (L4440:: Luc)
mml-1 (ok849)	*cco-1*	32	10.34%	84/120	55	3.77%	NS	vs. N2 (cco-1)
mxl-2 (tm1516)	*cco-1*	24	-17.24%	94/120	36	-32.08%	<0.0001	vs. N2 (cco-1)

N2	L4440::Luc	16		66/120	26			
mml-1 (ok849)	L4440::Luc	14	-12.50%	60/120	19	-26.92%	<0.0001	vs. N2 (L4440:: Luc)
mxl-2 (tm1516)	L4440::Luc	16	0.00%	94/120	19	-26.92%	0.0013	vs. N2 (L4440:: Luc)
N2	*cco-1*	33	106.25%	82/120	47	80.77%	<0.0001	vs. N2 (L4440:: Luc)
mml-1 (ok849)	*cco-1*	29	-12.12%	89/120	43	-8.51%	NS	vs. N2 (cco-1)
mxl-2 (tm1516)	*cco-1*	22	-33.33%	94/120	38	-19.15%	<0.0001	vs. N2 (cco-1)

***isp-1* lifespan**

mml-1 is required for *isp-1* longevity (2 out of 3).

N2	26		97/120	35			
mml-1 (ok849)	29	11.54%	92/120	37	5.71%	NS	vs. N2
mxl-2 (tm1516)	15	-42.31%	99/120	29	-17.14%	<0.0001	vs. N2
isp-1(qm150)	42	61.54%	44/120	64	82.86%	<0.0001	vs. N2
isp-1(qm150); mml-1 (ok849)	26	-38.10%	63/120	59	-7.81%	<0.0001	vs. *isp-1(qm150)*
isp-1(qm150); mxl-2 (tm1516)	22	-47.62%	60/120	47	-26.56%	<0.0001	vs. *isp-1(qm150)*

N2	22		53/120	31			
mml-1 (ok849)	22	0.00%	64/120	31	0.00%	NS	vs. N2
mxl-2 (tm1516)	22	0.00%	58/120	34	9.68%	0.0263	vs. N2
isp-1(qm150)	24	9.09%	22/120	56	80.65%	0.0176	vs. N2
isp-1(qm150); mml-1 (ok849)	27	12.50%	36/120	52	-7.14%	NS	vs. *isp-1(qm150)*
isp-1(qm150); mxl-2 (tm1516)	22	-8.33%	44/120	48	-14.29%	0.0183	vs. *isp-1(qm150)*

N2	23		44/120	35			
mml-1 (ok849)	21	-8.70%	75/120	28	-20.00%	0.0003	vs. N2
mxl-2 (tm1516)	21	-8.70%	54/120	35	0.00%	NS	vs. N2
isp-1(qm150)	40	73.91%	29/120	58	65.71%	<0.0001	vs. N2
isp-1(qm150); mml-1 (ok849)	26	-35.00%	37/120	58	0.00%	0.0326	vs. *isp-1(qm150)*
isp-1(qm150); mxl-2 (tm1516)	21	-47.50%	49/120	42	-27.59%	<0.0001	vs. *isp-1(qm150)*

***mml-1* overexpression lifespan**

mml-1 overexpression modestly extends N2 lifespan (2 out of 3).

N2	22		84/120	33			
wgIs198 [mml-1::TY1::EGFP::3xFLAG(92C12) + unc-119(+)]	29	31.82%	36/120	40	21.21%	0.0299	vs. N2

N2	22		87/120	36			
wgIs198 [mml-1::TY1::EGFP::3xFLAG(92C12) + unc-119(+)]	17	-22.73%	57/120	36	0.00%	NS	vs. N2

N2	22		90/140	36			
wgIs198 [mml-1::TY1::EGFP::3xFLAG(92C12) + unc-119(+)]	25	13.64%	110/140	43	19.44%	0.0010	vs. N2

***mml-1* overexpression lifespan**

mml-1 overexpression modestly extends N2 lifespan (1 out of 3).

N2	22		84/120	36			
dhEx966 (mml-1p::mml-1::GFP, myo-2p::mCherry)	19	-13.64%	90/120	33	-8.33%	NS	vs. N2

dhEx967(mml-1p::mml-1::GFP, myo-2p::mCherry)	22	0.00%	87/120	29	-19.44%	NS	vs. N2

N2	20		87/120	27			
dhEx966 (mml-1p::mml-1::GFP, myo-2p::mCherry)	22	10.00%	98/120	31	14.81%	<0.0001	vs. N2
dhEx967(mml-1p::mml-1::GFP, myo-2p::mCherry)	25	25.00%	91/120	36	33.33%	<0.0001	vs. N2

N2	22		84/120	33			
dhEx966 (mml-1p::mml-1::GFP, myo-2p::mCherry)	22	0.00%	76/120	33	0.00%	NS	vs. N2
dhEx967(mml-1p::mml-1::GFP, myo-2p::mCherry)	22	0.00%	78/120	33	0.00%	NS	vs. N2

***mml-1* overexpression lifespan in *glp-1* background**

mml-1 overexpression did not further extend *glp-1* longevity.

N2	26		93/120	37			
glp-1 (e2141)	37	42.31%	76/125	52	40.54%	<0.0001	vs. N2
glp-1 (e2141);wgIs198 [mml-1::TY1::EGFP::3xFLAG(92C12) + unc-119(+)]	40	8.11%	53/71	58	11.54%	NS	vs. *glp-1*

N2	24		104/120	40			
glp-1 (e2141)	37	54.17%	105/120	54	35.00%	<0.0001	vs. N2
glp-1 (e2141);wgIs198 [mml-1::TY1::EGFP::3xFLAG(92C12) + unc-119(+)]	33	-10.81%	85/120	50	-7.41%	NS	vs. *glp-1*

N2	22		113/120	38			
glp-1 (e2141)	35	59.09%	104/120	52	36.84%	<0.0001	vs. N2
glp-1 (e2141);wgIs198 [mml-1::TY1::EGFP::3xFLAG(92C12) + unc-119(+)]	31	-11.43%	99/120	59	13.46%	0.0984	vs. *glp-1*

***mml-1* rescue lifespan**

mml-1 overexpression rescues the shortevity of *glp-1mml-1*.

N2	21		56/120	31			
glp-1 (e2141)	35	66.67%	75/120	51	64.52%	<0.0001	vs. N2
glp-1 (e2141) mml-1 (ok849)	19	-45.71%	97/120	26	-49.02%	<0.0001	vs. *glp-1*

glp-1 (e2141) mml-1 (ok849);wgIs198 [mml-1::TY1::EGFP::3xFLAG(92C12) + unc-119(+)]	35	0.00%	68/120	51	0.00%	NS	vs. *glp-1*

N2	25		47/120	36			
glp-1 (e2141)	39	56.00%	61/120	55	52.78%	<0.0001	vs. N2
glp-1 (e2141) mml-1 (ok849)	18	-53.85%	61/120	25	-54.55%	<0.0001	vs. *glp-1*
glp-1 (e2141) mml-1 (ok849);wgIs198 [mml-1::TY1::EGFP::3xFLAG(92C12) + unc-119(+)]	32	-17.95%	95/120	55	0.00%	0.03891	vs. *glp-1*

N2	30		93/150	42			
glp-1 (e2141)	39	30.00%	87/150	70	66.67%	<0.0001	vs. N2
glp-1 (e2141) mml-1 (ok849)	18	-53.85%	56/150	24	-65.71%	<0.0001	vs. *glp-1*
glp-1 (e2141) mml-1 (ok849);wgIs198 [mml-1::TY1::EGFP::3xFLAG(92C12) + unc-119(+)]	30	-23.08%	104/150	68	-2.86%	0,00575	vs. *glp-1*

***mml-1* rescue lifespan**

mml-1 overexpression rescues the shortevity of *glp-1mml-1*.

N2	24		88/120	35			
glp-1 (e2141)	31	29.17%	105/120	45	28.57%	<0.0001	vs. N2
glp-1 (e2141) mml-1 (ok849)	19	-38.71%	88/120	26	-42.22%	<0.0001	vs. *glp-1*
glp-1 (e2141) mml-1 (ok849); dhEx966 (mml-1p::mml-1::GFP, myo-2p::mCherry)	26	-16.13%	89/120	41	-8.89%	0.01782	vs. *glp-1*
glp-1 (e2141) mml-1 (ok849); dhEx967(mml-1p::mml-1::GFP, myo-2p::mCherry)	26	-16.13%	94/120	42	-6.67%	0.01561	vs. *glp-1*

N2	23		94/120	35			
glp-1 (e2141)	32	39.13%	89/120	46	31.43%	<0.0001	vs. N2
glp-1 (e2141) mml-1 (ok849)	16	-50.00%	57/120	23	-50.00%	<0.0001	vs. *glp-1*
glp-1 (e2141) mml-1 (ok849); dhEx966 (mml-1p::mml-1::GFP, myo-2p::mCherry)	27	-15.63%	70/120	40	-13.04%	0.0186	vs. *glp-1*
glp-1 (e2141) mml-1 (ok849); dhEx967(mml-1p::mml-1::GFP, myo-2p::mCherry)	27	-15.63%	80/120	44	-4.35%	0.0106	vs. *glp-1*

***TOR* lifespan**

mml-1 and *mxl-2* are required for TOR longevity.

N2	L4440::Luc	23		72/120	32			
mml-1 (ok849)	L4440::Luc	17	-26.09%	60/120	28	-12.50%	<0.0001	vs. N2 (L4440::Luc)
mxl-2 (tm1516)	L4440::Luc	18	-21.74%	92/120	32	0.00%	0.0035	vs. N2 (L4440::Luc)
N2	TOR	25	8.70%	52/120	35	9.38%	<0.0001	vs. N2 (L4440::Luc)
mml-1 (ok849)	TOR	17	-32.00%	58/120	25	-28.57%	<0.0001	vs. N2 (TOR)
mxl-2 (tm1516)	TOR	18	-28.00%	86/120	28	-20.00%	<0.0001	vs. N2 (TOR)

N2	L4440::Luc	26		46/120	31			
mml-1 (ok849)	L4440::Luc	19	-26.92%	53/120	33	6.45%	<0.0001	vs. N2 (L4440::Luc)
mxl-2 (tm1516)	L4440::Luc	17	-34.62%	80/120	28	-9.68%	<0.0001	vs. N2 (L4440::Luc)
N2	TOR	28	7.69%	48/120	41	32.26%	0.0003	vs. N2 (L4440::Luc)
mml-1 (ok849)	TOR	17	-39.29%	55/120	21	-48.78%	<0.0001	vs. N2 (TOR)
mxl-2 (tm1516)	TOR	14	-50.00%	113/120	21	-48.78%	<0.0001	vs. N2 (TOR)

N2	L4440::Luc	23		63/120	30			
mml-1 (ok849)	L4440::Luc	16	-30.43%	41/120	25	-16.67%	<0.0001	vs. N2 (L4440::Luc)
mxl-2 (tm1516)	L4440::Luc	18	-21.74%	80/120	25	-16.67%	<0.0001	vs. N2 (L4440::Luc)
N2	TOR	28	21.74%	43/120	35	16.67%	0.0007	vs. N2 (L4440::Luc)
mml-1 (ok849)	TOR	16	-42.86%	36/120	21	-40.00%	<0.0001	vs. N2 (TOR)
mxl-2 (tm1516)	TOR	16	-42.86%	61/120	21	-40.00%	<0.0001	vs. N2 (TOR)

***TOR* lifespan**

TOR knockdown fails to rescue shortevity of *glp-1mml-1* and *glp-1mxl-2*.

N2	L4440::Luc	25		53/120	32			
glp-1 (e2141)	L4440::Luc	30	20.00%	77/120	44	37.50%	<0.0001	vs. N2 (L4440::Luc)
glp-1 (e2141) mml-1 (ok849)	L4440::Luc	15	-50.00%	58/120	21	-52.27%	<0.0001	vs. *glp-1* (L4440::Luc)
glp-1 (e2141) mxl-2 (tm1516)	L4440::Luc	15	-50.00%	108/120	23	-47.73%	<0.0001	vs. *glp-1* (L4440::Luc)
N2	TOR	32	28.00%	53/120	44	37.50%	<0.0001	vs. N2 (L4440::Luc)
glp-1 (e2141)	TOR	28	-6.67%	69/120	39	-11.36%	0.0032	vs. *glp-1* (L4440::Luc)
glp-1 (e2141) mml-1 (ok849)	TOR	17	-39.29%	43/120	21	-46.15%	<0.0001	vs. *glp-1* (TOR)
glp-1 (e2141) mxl-2 (tm1516)	TOR	15	-46.43%	94/120	21	-46.15%	<0.0001	vs. *glp-1* (TOR)

glp-1 (e2141)	L4440::Luc	28		48/120	36			
glp-1 (e2141) mml-1 (ok849)	L4440::Luc	19	-32.14%	53/120	21	-41.67%	<0.0001	vs. *glp-1* (L4440::Luc)

glp-1 (e2141) mxl-2 (tm1516)	L4440::Luc	14	-50.00%	112/120	23	-36.11%	<0.0001	vs. *glp-1* (L4440:: Luc)
glp-1 (e2141)	TOR	26	-7.14%	50/120	36	0.00%	NS	vs. *glp-1* (L4440:: Luc)
glp-1 (e2141) mml-1 (ok849)	TOR	14	-46.15%	79/120	19	-47.22%	<0.0001	vs. *glp-1* (TOR)
glp-1 (e2141) mxl-2 (tm1516)	TOR	14	-46.15%	102/120	19	-47.22%	<0.0001	vs. *glp-1* (TOR)

glp-1 (e2141)	L4440::Luc	26		78/120	36			
glp-1 (e2141) mml-1 (ok849)	L4440::Luc	17	-34.62%	49/120	19	-47.22%	<0.0001	vs. *glp-1* (L4440:: Luc)
glp-1 (e2141) mxl-2 (tm1516)	L4440::Luc	15	-42.31%	93/120	17	-52.78%	<0.0001	vs. *glp-1* (L4440:: Luc)
glp-1 (e2141)	TOR	27	3.85%	67/120	36	0.00%	0.0585	vs. *glp-1* (L4440:: Luc)
glp-1 (e2141) mml-1 (ok849)	TOR	17	-37.04%	35/120	19	-47.22%	<0.0001	vs. *glp-1* (TOR)
glp-1 (e2141) mxl-2 (tm1516)	TOR	15	-44.44%	86/120	20	-44.44%	<0.0001	vs. *glp-1* (TOR)

HLH-30 overexpression lifespan

HLH-30::GFP overexpression does not rescue the shortevity of *glp-1mml-1* and *glp-1mxl-2*.

N2		25		49/120	39			
glp-1 (e2141)		39	56.00%	83/120	53	35.90%	<0.0001	vs. N2
glp-1 (e2141) mml-1 (ok849)		22	-43.59%	82/120	27	-49.06%	<0.0001	vs. *glp-1*
glp-1 (e2141) mxl-2 (tm1516)		20	-48.72%	96/120	27	-49.06%	<0.0001	vs. *glp-1*
glp-1(e2141) mml-1(ok849);sqIs19[hlh-30p::hlh-30::GFP + rol-6(su1006)]		18	-53.85%	104/120	27	-49.06%	<0.0001	vs. *glp-1*
glp-1(e2141) mxl-2 (tm1516); sqIs19[hlh-30p::hlh-30::GFP + rol-6(su1006)]		18	-53.85%	106/120	32	-39.62%	<0.0001	vs. *glp-1*

N2		25		91/125	38			
glp-1 (e2141)		34	36.00%	87/125	48	26.32%	<0.0001	vs. N2
glp-1 (e2141) mml-1 (ok849)		16	-52.94%	76/125	24	-50.00%	<0.0001	vs. *glp-1*
glp-1 (e2141) mxl-2 (tm1516)		17	-50.00%	104/125	29	-39.58%	<0.0001	vs. *glp-1*
glp-1(e2141) mml-1(ok849);sqIs19[hlh-30p::hlh-30::GFP + rol-6(su1006)]		16	-52.94%	69/125	24	-50.00%	<0.0001	vs. *glp-1*
glp-1(e2141) mxl-2(tm1516); sqIs19[hlh-30p::hlh-30::GFP + rol-6(su1006)]		16	-52.94%	92/125	25	-47.92%	<0.0001	vs. *glp-1*

lars-1 **knockdown lifespan**

lars-1 knockdown fails tor rescue the shortevity of *glp-1mml-1* and *glp-1mxl-2*

N2	L4440::Luc	25		71/120	32			
glp-1 (e2141)	L4440::Luc	28	12.00%	67/120	38	18.75%	<0.0001	vs. N2 (L4440:: Luc)

glp-1 (e2141) mml-1 (ok849)	L4440::Luc	17	-39.29%	49/120	19	-50.00%	<0.0001	vs. *glp-1* (L4440:: Luc)
N2	lars-1	19	-24.00%	52/120	38	18.75%	0.0961	vs. N2 (L4440:: Luc)
glp-1 (e2141)	lars-1	25	-10.71%	54/120	33	-13.16%	<0.0001	vs. *glp-1* (L4440:: Luc)
glp-1 (e2141) mml-1 (ok849)	lars-1	17	-32.00%	44/120	22	-33.33%	<0.0001	vs. *glp-1* (lars-1)

N2	L4440::Luc	20		77/125	26			
glp-1 (e2141)	L4440::Luc	26	30.00%	78/125	36	38.46%	<0.0001	vs. N2 (L4440:: Luc)
glp-1 (e2141) mxl-2 (tm1516)	L4440::Luc	15	-42.31%	93/125	17	-52.78%	<0.0001	vs. *glp-1* (L4440:: Luc)
N2	lars-1	15	-25.00%	60/125	27	3.85%	<0.0001	vs. N2 (L4440:: Luc)
glp-1 (e2141)	lars-1	26	0.00%	73/125	30	-16.67%	NS	vs. *glp-1* (L4440:: Luc)
glp-1 (e2141) mxl-2 (tm1516)	lars-1	15	-42.31%	56/125	26	-13.33%	<0.0001	vs. *glp-1* (lars-1)

***raga-1* lifespan**

raga-1 deletion fails to rescue the *glp-1mxl-2* shortevity.

N2		22		56/120	32			
mxl-2 (tm1516)		13	-40.91%	71/120	32	0.00%	0.0354	vs. N2
raga-1 (ok701)		27	22.73%	60/120	39	21.88%	<0.0001	vs. N2
glp-1 (e2141)		36	63.64%	75/120	52	62.50%	<0.0001	vs. N2
glp-1 (e2141) mxl-2 (tm1516)		18	-50.00%	105/120	25	-51.92%	<0.0001	vs. *glp-1*
glp-1(e2141);raga-1(ok701)		32	-11.11%	53/85	49	-5.77%	0.0006	vs. *glp-1*
glp-1 (e2141) mxl-2 (tm1516);raga-1(ok701)		20	-44.44%	50/120	27	-48.08%	<0.0001	vs. *glp-1*

N2		27		69/120	34			
mxl-2 (tm1516)		18	-33.33%	101/120	34	0.00%	<0.0001	vs. N2
raga-1 (ok701)		27	0.00%	101/120	38	11.76%	NS	vs. N2
glp-1 (e2141)		41	51.85%	58/120	59	73.53%	<0.0001	vs. N2
glp-1 (e2141) mxl-2 (tm1516)		21	-48.78%	89/120	29	-50.85%	<0.0001	vs. *glp-1*
glp-1(e2141);raga-1(ok701)		34	-17.07%	84/120	41	-30.51%	<0.0001	vs. *glp-1*
glp-1 (e2141) mxl-2 (tm1516);raga-1(ok701)		15	-63.41%	70/120	18	-69.49%	<0.0001	vs. *glp-1*

***glp-1* lifespan**

hlh-30 is required for *glp-1* longevity.

N2		24		40/120	31			
hlh-30 (tm1978)		17	-29.17%	53/120	26	-16.13%	0.0001	vs. N2
glp-1 (e2141)		26	8.33%	67/120	43	38.71%	0.0146	vs. N2
glp-1 (e2141); hlh-30 (tm1978)		24	-7.69%	105/120	33	-23.26%	<0.0001	vs. *glp-1*

N2		26		41/120	41			
hlh-30 (tm1978)		26	0.00%	33/120	33	-19.51%	0.0028	vs. N2

glp-1 (e2141)		35	34.62%	50/120	50	21.95%	<0.0001	vs. N2
glp-1 (e2141); hlh-30 (tm1978)		19	-45.71%	30/120	30	-40.00%	<0.0001	vs. *glp-1*

N2		24		83/150	45			
hlh-30 (tm1978)		20	-16.67%	67/150	30	-33.33%	<0.0001	vs. N2
glp-1 (e2141)		41	70.83%	71/150	62	37.78%	<0.0001	vs. N2
glp-1 (e2141); hlh-30 (tm1978)		24	-41.46%	140/150	30	-51.61%	<0.0001	vs. *glp-1*

***glp-1* lifespan**

mdl-1/mxl-1 is required for *glp-1* longevity

N2		26		91/120	37			
mdl-1 (tm311)		16	-38.46%	93/120	26	-29,73%	<0.0001	vs. N2
mxl-1 (tm1530)		26	0.00%	96/120	35	-5.41%	NS	vs. N2
glp-1 (e2141)		35	34.62%	100/120	65	75.68%	<0.0001	vs. N2
glp-1 (e2141);mdl-1 (tm311)		11	-68.57%	64/120	33	-49.23%	<0.0001	vs. *glp-1*
glp-1 (e2141);mxl-1 (tm1530)		19	-45.71%	101/120	37	-43.08%	<0.0001	vs. *glp-1*

N2		24		104/120	40			
mdl-1 (tm311)		15	-37.50%	97/120	26	-35.00%	<0.0001	vs. N2
mxl-1 (tm1530)		24	0.00%	94/120	38	-5.00%	NS	vs. N2
glp-1 (e2141)		37	54.17%	105/120	54	35.00%	<0.0001	vs. N2
glp-1 (e2141);mdl-1 (tm311)		12	-67.57%	99/120	26	-51.85%	<0.0001	vs. *glp-1*
glp-1 (e2141);mxl-1 (tm1530)		12	-65.7%	43/120	37	-31.48%	<0.0001	vs. *glp-1*

N2		23		94/122	32			
mdl-1 (tm311)		13	-43.48%	109/150	32	0.00%	<0.0001	vs. N2
mxl-1 (tm1530)		20	-13.04%	83/120	32	0.00%	NS	vs. N2
glp-1 (e2141)		32	39.13%	89/160	48	50.00%	<0.0001	vs. N2
glp-1 (e2141);mdl-1 (tm311)		11	-65.63%	99/121	25	-47.92%	<0.0001	vs. *glp-1*
glp-1 (e2141);mxl-1 (tm1530)		11	-59.38%	129/160	32	-33.33%	<0.0001	vs. *glp-1*

Candidate lifespan from RNA-seq

RNAi treatment from egg onward.

N2	L4440::Luc	21		70/120	32			
glp-1 (e2141)	L4440::Luc	32	52.38%	90/120	47	46.88%	<0.0001	vs. N2 (L4440:: Luc)
N2	*fat-5*	17	-19.05%	60/120	27	-15.63%	<0.0001	vs. N2 (L4440:: Luc)
glp-1 (e2141)	*fat-5*	12	-62.50%	88/120	21	-55.32%	<0.0001	vs. glp-1 (L4440:: Luc)
N2	*swt-1*	25	19.05%	78/120	34	6.25%	NS	vs. N2 (L4440:: Luc)
glp-1 (e2141)	*swt-1*	25	-21.88%	87/120	41	-12.77%	<0.0001	vs. glp-1 (L4440:: Luc)
N2	*ugt-53*	21	0.00%	83/120	32	-31.91%	NS	vs. N2 (L4440:: Luc)
glp-1 (e2141)	*ugt-53*	25	-21.88%	82/120	36	-23.40%	<0.0001	vs. glp-1 (L4440:: Luc)

RNAi treatment during adulthood

N2	L4440::Luc	22		83/120	36			
glp-1 (e2141)	L4440::Luc	31	40.91%	82/120	47	30.56%	<0.0001	vs. N2 (L4440::Luc)
N2	*fat-5*	17	-22.73%	101/120	29	-19.44%	<0.0001	vs. N2 (L4440::Luc)
glp-1 (e2141)	*fat-5*	24	-22.58%	108/120	43	-8.51%	<0.0001	vs. glp-1 (L4440::Luc)
N2	*swt-1*	24	9.09%	111/120	33	-8.33%	NS	vs. N2 (L4440::Luc)
glp-1 (e2141)	*swt-1*	29	-6.45%	88/120	38	-19.15%	0.0265	vs. glp-1 (L4440::Luc)
N2	*ugt-53*	22	0.00%	77/120	31	-13.89%	NS	vs. N2 (L4440::Luc)
glp-1 (e2141)	*ugt-53*	33	6.45%	83/120	43	-8.51%	NS	vs. glp-1 (L4440::Luc)

fat-5 **mutant lifespan**

fat-5 deletion suppresses *glp-1* longevity.

N2	21		49/120	32			
fat-5 (tm420)	14	-33.33%	62/120	35	9.38%	NS	vs. N2
glp-1 (e2141)	32	52.38%	71/120	46	43.75%	<0.0001	vs. N2
glp-1 (e2141); fat-5 (tm420)	18	-43.75%	114/120	39	-15.22%	<0.0001	vs. *glp-1*

N2	20		51/120	31			
fat-5 (tm420)	17	-15.00%	55/120	29	-6.45%	NS	vs. N2
glp-1 (e2141)	31	55.00%	71/120	45	45.16%	<0.0001	vs. N2
glp-1 (e2141); fat-5 (tm420)	10	-67.74%	108/120	38	-15.56%	<0.0001	vs. *glp-1*

mdl-1**::GFP rescue lifespan**

mdl-1 overexpression patially rescue the shortevity of *glp-1mml-1* and *glp-1mxl-2*

N2	25		62/120	36			
glp-1(e2141)	34	36.00%	92/120	53	47.22%	<0.0001	vs. N2
glp-1(e2141) mml-1(ok849)	18	-47.06%	64/120	22	-58.49%	<0.0001	vs. *glp-1*
glp-1(e2141) mxl-2(tm1516)	18	-47.06%	94/120	25	-52.83%	<0.0001	vs. *glp-1*
glp-1(e2141); wgIs106[mdl-1::TY1::EGFP::3xFLAG + unc-119(+)]	36	5.88%	86/120	55	3.77%	NS	vs. *glp-1*
glp-1(e2141) mml-1(ok849); wgIs106[mdl-1::TY1::EGFP::3xFLAG + unc-119(+)]	25	-26.47%	100/120	43	-18.87%	<0.0001	vs. *glp-1*
glp-1(e2141) mxl-2(tm1516); wgIs106[mdl-1::TY1::EGFP::3xFLAG + unc-119(+)]	27	-20.59%	64/120	43	-18.87%	<0.0001	vs. *glp-1*

N2	18		94/120	35			
glp-1(e2141)	40	122.22%	102/120	51	45.71%	<0.0001	vs. N2

glp-1(e2141) mml-1(ok849)	14	-65.00%	94/120	23	-54.90%	<0.0001	vs. *glp-1*
glp-1(e2141) mxl-2(tm1516)	18	-5.00%	104/120	23	-54.90%	<0.0001	vs. *glp-1*
glp-1(e2141); wgIs106[mdl-1::TY1::EGFP::3xFLAG + unc-119(+)]	42	5.00%	59/120	47	-7.84%	NS	vs. *glp-1*
glp-1(e2141) mml-1(ok849); wgIs106[mdl-1::TY1::EGFP::3xFLAG + unc-119(+)]	23	-42.50%	105/120	35	-31.37%	<0.0001	vs. *glp-1*
glp-1(e2141) mxl-2(tm1516); wgIs106[mdl-1::TY1::EGFP::3xFLAG + unc-119(+)]	23	-42.50%	106/120	37	-27.45%	<0.0001	vs. *glp-1*

***mdl-1* overexpression lifespan**

mdl-1 overexpression extends wild type lifespan.

N2	18		94/120	35			
wgIs106[mdl-1::TY1::EGFP::3xFLAG + unc-119(+)]	26	44.44%	78/120	44	25.71%	<0.0001	vs. N2

N2	22		72/120	34			
wgIs106[mdl-1::TY1::EGFP::3xFLAG + unc-119(+)]	27	22.73%	59/120	42	23.53%	0.0009	vs. N2

* no. of dead animals/total no. of animals analyzed

P values were calculated by the Mantel-Cox Log Rank test.

Supplementary Table 2: Strain Lists.

Strains	Genotype	Experiments
	N2 (WT)	
AA3234	*mml-1(ok849)III*	The strain was outcrossed 3 times to N2
AA3235	*mxl-2(tm1516)III*	The strain was outcrossed 3 times to N2
AA3561	*glp-1(e2141ts)III*	
AA3232	*glp-1(e2141) mml-1(ok849)III*	
AA3560	*glp-1(e2141ts) mxl-2(tm1516)III*	
CB1370	*daf-2(e1370)III*	
AA3407	*daf-2 (e1370) mml-1 (ok849)III*	
AA3408	*daf-2(e1370) mxl-2(tm1516)III*	
MQ887	*isp-1(qm150)IV*	
AA3409	*mml-1(ok849)III; isp-1 (qm150)IV*	
AA3410	*mxl-2(tm1516)III; isp-1(qm150)IV*	
AA3301	*dhEx966 (mml-1p::mml-1::GFP, myo-2p::mCherry)*	used for MML-1::GFP quantification and lifespan
AA3302	*dhEx967(mml-1p::mml-1::GFP, myo-2p::mCherry)*	used for MML-1::GFP quantification and lifespan
AA3383	*glp-1; dhEx966(mml-1p::mml-1::GFP, myo-2p::mCherry)*	used for MML-1::GFP quantification and lifespan
AA3384	*glp-1; dhEx967(mml-1p::mml-1::GFP, myo-2p::mCherry)*	used for MML-1::GFP quantification and lifespan
OP198	*wgIs198 [mml-1::TY1::EGFP::3xFLAG(92C12) + unc-119(+)]*	used for MML-1::GFP quantification and lifespan
AA3771	*glp-1(e2141)III; wgIs198 [mml-1::TY1::EGFP::3xFLAG(92C12) + unc-119(+)]*	used for MML-1::GFP quantification and lifespan
AA3429	*glp-1(e2141) mml-1(ok849)III;dhEx966(mml-1a::GFP, myo-2p::mCherry)*	used for rescue lifespan
AA3402	*glp-1(e2141) mml-1(ok849)III; dhEx967(mml-1a::GFP, myo-2p::mCherry)*	used for rescue lifespan
AA3762	*glp-1(e2141) mml-1(ok849) III; wgIs198[mml-1::TY1::EGFP::3xFLAG(92C12) + unc-119(+)]*	used for rescue lifespan
AA3848	*N2; dhEx1035(mxl-2::GFP, myo-2p::mCherry)*	MXL-2::GFP quantification
AA3911	*glp-1(e2141)III; dhEx1035(mxl-2p::mxl-2::GFP; myo-2p::mCherry)*	MXL-2::GFP quantification
AA3923	*glp-1(e2141) mxl-2(tm1516)III; dhEx1035(mxl-2::GFP, myo-2p::mCherry)*	
MAH235	*sqIs19[hlh-30p::hlh-30::GFP + rol-6(su1006)]*	HLH-30::GFP nuclear localization assay
AA3572	*glp-1(e2141)III; sqIs19[hlh-30p::hlh-30::GFP + rol-6(su1006)]*	HLH-30::GFP nuclear localization assay
AA3570	*glp-1(e2141) mml-1(ok849)III; sqIs19[hlh-30p::hlh-30::GFP + rol-6(su1006)]*	HLH-30::GFP nuclear localization assay
AA3571	*glp-1(e2141) mxl-2(tm1516)III; sqIs19[hlh-30p::hlh-30::GFP + rol-6(su1006)]*	HLH-30::GFP nuclear localization assay
AA4036	*eat-2(ad465)II; sqIs19[hlh-30p::hlh-30::GFP + rol-6(su1006)]*	HLH-30::GFP nuclear localization assay
AA3961	*daf-2 (e1370)III; sqIs19[hlh-30p::hlh-30::GFP + rol-6(su1006)]*	HLH-30::GFP nuclear localization assay
DA2123	*adIs2122(lgg-1::GFP; rol-6(su1006))*	LGG-1::GFP puncta counting
AA3256	*glp-1(e2141)III; adIs2122[lgg-1::GFP + rol-6(su1006)]*	LGG-1::GFP puncta counting
AA3299	*glp-1(e2141) mml-1(ok849)III;adIs2122(lgg-1::GFP; rol-6(su1006))*	LGG-1::GFP puncta counting
AA3386	*glp-1(e2141) mxl-2 (tm1516)III; adIs2122[lgg-1::GFP + rol-6(su1006)]*	LGG-1::GFP puncta counting
RT258	*unc-119(ed3) III; pwIs50pwIs50 [lmp-1::GFP + Cbr-unc-119(+)].*	LMP-1::GFP Western Blotting
AA3575	*glp-1(e2141)III; pwIs50pwIs50[lmp-1::GFP + Cbr-unc-119(+)].*	LMP-1::GFP Western Blotting
AA3573	*glp-1(e2141); mml-1(ok849)III; pwIs50pwIs50 [lmp-1::GFP + Cbr-unc-119(+)].*	LMP-1::GFP Western Blotting
AA3574	*glp-1(e2141); mxl-2(tm1516)III; pwIs50pwIs50 [lmp-1::GFP + Cbr-unc-119(+)].*	LMP-1::GFP Western Blotting
VIG9	*unc119(ed3)III; Is[unc-119(+); Plgg-2::gfp::lgg-2]*	LGG-2::GFP puncta counting
AA3579	*glp-1(e2141) III; Is[unc-119(+); Plgg-2::gfp::lgg-2]*	LGG-2::GFP puncta counting
AA3577	*glp-1(e2141) mml-1(ok849) III; Is[unc-119(+); Plgg-2::gfp::lgg-2]*	LGG-2::GFP puncta counting
AA3578	*glp-1(e2141) mxl-2(tm1516) III; Is[unc-119(+); Plgg-2::gfp::lgg-2]*	LGG-2::GFP puncta counting
HZ589	*bpIs151(T12G3.1::gfp;unc-76) IV; him-5(e1490) V*	SQST-1/p62::GFP

		quantification and Western Blotting
AA3564	*glp-1(e2141)III;bpIs151(T12G3.1::gfp;unc-76)IV; him-5(e1490)V*	SQST-1/p62::GFP quantification and Western Blotting
AA3562	*glp-1(e2141) mml-1(ok849)III;bpIs151(T12G3.1::gfp;unc-76)IV; him-5(e1490)V*	SQST-1/p62::GFP quantification and Western Blotting
AA3563	*glp-1(e2141) mxl-2(tm1516)III;bpIs151(T12G3.1::gfp;unc-76)IV; him-5(e1490)V*	SQST-1/p62::GFP quantification and Western Blotting
AA3826	*mml-1(ok849)III; bpIs151(T12G3.1::gfp;unc-76)IV; him-5(e1490)V*	SQST-1/p62::GFP Western Blotting
AA3827	*mxl-2(tm1516)III; bpIs151(T12G3.1::gfp;unc-76) IV; him-5(e1490) V*	SQST-1/p62::GFP Western Blotting
AA3776	*raga-1(ok701) II*	used for rescue lifespan
AA3732	*raga-1(ok701) II; glp-1(e2141) III*	used for rescue lifespan
AA3734	*raga-1 (ok701) II; glp-1(e2141) mxl-2(tm1516) III*	used for rescue lifespan
AA3837	*N2; dhEx1031(lars-1::GFP, myo-2p::mCherry)*	LARS-1::GFP quantification
AA3863	*glp-1(e2141)III; dhEx1031(lars-1::GFP, myo-2p::mCherry)*	LARS-1::GFP quantification
AA3864	*glp-1(e2141) mml-1(ok849)III; dhEx1031(lars-1::GFP, myo-2p::mCherry)*	LARS-1::GFP quantification
AA3865	*glp-1(e2141) mxl-2(tm1516)III; dhEx1031(lars-1::GFP, myo-2p::mCherry)*	LARS-1::GFP quantification
AA3658	*hlh-30 (tm1978) IV*	The strain was outcrossed 6 times to N2
AA3855	*glp-1(e2141ts); hlh-30 (tm1978) IV*	
AA3840	*mml-1(ok849) III; hlh-30(tm1978) IV*	
AA3841	*mxl-2(tm1516) III; hlh-30(tm1978) IV*	
AA3858	*glp-1(e2141ts) mml-1(ok849) III; hlh-30(tm1978) IV*	
AA3859	*glp-1(e2141ts) mxl-2(tm1516) III; hlh-30(tm1978) IV*	
OP106	*unc-119(ed3) III; wgIs106[mdl-1::TY1::EGFP::3xFLAG + unc-119(+)]*	The strain was outcrossed one time to N2
AA3773	*glp-1(e2141) III; wgIs106[mdl-1::TY1::EGFP::3xFLAG + unc-119(+)]*	
AA3774	*glp-1(e2141) mml-1(ok849) III; wgIs106[mdl-1::TY1::EGFP::3xFLAG + unc-119(+)]*	
AA3775	*glp-1(e2141) mxl-2(tm1516) III; wgIs106[mdl-1::TY1::EGFP::3xFLAG + unc-119(+)]*	
AA3774	*glp-1(e2141) mml-1(ok849)III; wgIs106[mdl-1::TY1::EGFP::3xFLAG + unc-119(+)]*	used for rescue lifespan
AA3775	*glp-1(e2141) mxl-2(tm1516)III; wgIs106[mdl-1::TY1::EGFP::3xFLAG + unc-119(+)]*	used for rescue lifespan
AA3372	*mdl-1(tm311) X*	The strain was outcrossed 3 times to N2
AA3415	*glp-1(e2141) III; mdl-1(tm311) X*	
AA3373	*mxl-1(tm1530)V*	outcrossed 3 times to N2
AA3416	*glp-1(e2141)III; mxl-1(tm1530)V*	
BX107	*fat-5(tm420)*	outcrossed 3 times to N2
HGA8006	*glp-1(e2141)III; fat-5 (tm420)V*	outcrossed one time to N2

Supplementary Table 3: PCR Primers.

Name	Sequence	Purpose
mml-1 ok849 f2	TTCTGCTAGATAACTTACCACC	Genotyping
mml-1 ok849 r	GTCTTCACAACCACAGTATGCCACA	Genotyping
mxl-2 tm1516 f	TTGGTTGGCTGACAAATTTCAATTGGAGAA	Genotyping
mxl-2 tm1516 r	GCCGGACGGCTCGGCGAGCTCCCCATCGG	Genotyping
hlh-30 tm1978 f	CCGGATGAGGACTGAAACAT	Genotyping
hlh-30 tm1978 r	GGCTCAGGACACTCTGGAAG	Genotyping
daf-2 e1370 f	CCAGTGCTTCTGAATCGTCA	Genotyping
daf-2 e1370 r	TGGAGCTTCGGAGTTGTTCT	Genotyping
raga-1 ok701 f	AACTTCCTCGTCTCGCAAAA	Genotyping
raga-1 ok701 r	GTCTTCGGTGAATCCGTTGT	Genotyping
mdl-1 tm311 f	ACACTACCTCTCGCCGTCTGCCGCCT	Genotyping
mdl-1 tm311 r	AAATGGGGTAGGTGGAAGATTCAT	Genotyping
mxl-1 tm1530 f	ACATATTATTTATTAGATCTCTGTGA	Genotyping
mxl-1 tm1530 r	ATGTCTTTCAAATAATGTGCAAAGTCAG	Genotyping
fat-5 tm420 f	AGGCTTACCTCACCCGAAAT	Genotyping
fat-5 tm420 r	CAGAAACACGCCAGAGATCA	Genotyping
mml-1 f	CCGCTTGCGAGTACACTTTTG	qRT-PCR
mml-1 r	CGGCTCGGCGAGAATG	qRT-PCR
mxl-2 f	ATCACCAGAACGCTCCTGTC	qRT-PCR
mxl-2 r	TTTCCGGCTGTCGACTTGAA	qRT-PCR
lgg-1 f	ACCCAGACCGTATTCCAGTG	qRT-PCR
lgg-1 r	ACGAAGTTGGATGCGTTTTC	qRT-PCR
tts-1 f	CCGACACGTTTCAGACACAC	qRT-PCR
tts-1 r	GGTTTTACCCATTGACTCAACC	qRT-PCR
lgg-2 f	CTGCAAATTCCTAGTACCCGAG	qRT-PCR
lgg-2 r	CATAGAATTTGACACCATTGAGC	qRT-PCR
mdl-1 f	CTGCTCTCCTTCCGCCAAT	qRT-PCR
mdl-1 r	ATGAGTGCAAGGCGAATGG	qRT-PCR
swt-1 f	TGTCCAGCTCGCCCTCTT	qRT-PCR
swt-1 r	TTCTAACGGGCTCTTCTCATTCTC	qRT-PCR
atg-2 f	TCATCTGCAGCTGGTATAGTAGGTATG	qRT-PCR
atg-2 r	CGGCGAGACTTTGCACAA	qRT-PCR
epg-9 f	GACGCCGTTGAATGCATCT	qRT-PCR
epg-9 r	GGAACCTTCCAAGTGTTCTGTGA	qRT-PCR
atg-9 f	GCCAACACAAACGTGACAAGTC	qRT-PCR
atg-9 r	TCCGAAATGCCGTTTTCC	qRT-PCR
ctns-1 f	AAAGCGTCGTCGGGCTAA	qRT-PCR
ctns-1 r	ATGCACAAAATCCGACAAGGT	qRT-PCR
pyk-1 f	GGCGGTGCTTCTGCTGAA	qRT-PCR
pyk-1 r	GTGGATCAGTAGTCAAACGGATTG	qRT-PCR
tps-1 f	AAAGTGCCAACACCCGTTTT	qRT-PCR
tps-1 r	CCTCGTGGGAGCTGTTTCC	qRT-PCR
cpr-1 f	GCTGCCCACTCCGATGAA	qRT-PCR
cpr-1 r	GGGACAGAGGCGAGAACAAC	qRT-PCR
unc-51 f	TTAGGTCATGGAGCATTTGCAA	qRT-PCR
unc-51 r	GGCACGTCTGTGCGATCA	qRT-PCR
sams-1 f	AAGACCTGCAACGTGCTTGTT	qRT-PCR
sams-1 r	AACTCCGGCAGCAATTTCTG	qRT-PCR

pept-1 f	CCCAGTCGCTGCCAAGTG	qRT-PCR
pept-1 r	AAGCCCTCCAGTGACCATTTT	qRT-PCR
asm-3 f	CGCGCCATGCAACGA	qRT-PCR
asm-3 r	ACGGGCGTCGCAAAGATA	qRT-PCR
lars-1/R74.1 f	GATTGGCGTCGTTCCTTCA	qRT-PCR
lars-1/R74.1 r	CGGACGAATGAGTCGAAGTATG	qRT-PCR
mars-1 f	GTCGTTGTGCTTTGCAATCTG	qRT-PCR
mars-1 r	CCATGGCTCGGGACTCTACTC	qRT-PCR
fat-5 f	GGTCGGACTATATCAGCTTGTG	qRT-PCR
fat-5 r	CATGAGAGGGTGGCTTTGTAG	qRT-PCR
gpdh-1 f	GGAGCCCACGCGATCTC	qRT-PCR
gpdh-1 r	CCCCGTTCTCACAGCTTGA	qRT-PCR
cdc-42 f	CTGCTGGACAGGAAGATTACG	qRT-PCR
cdc-42 r	CTCGGACATTCTCGAATGAAG	qRT-PCR
ama-1 f	GGATGGAATGTGGGTTGAGA	qRT-PCR
ama-1 r	CGGATTCTTGAATTTCGCGC	qRT-PCR
SQSTM1 f	TTCTTTTCCCTCCGTGCTC	qRT-PCR
SQSTM1 r	GGATCCGAGTGTGAATTTCC	qRT-PCR
VPS11 f	ATCCAGAAACTTGCGGATCA	qRT-PCR
VPS11 r	ACCATCTCTACAGCAAGGGC	qRT-PCR
TFEB f	CCAGAAGCGAGAGCTCACAGAT	qRT-PCR
TFEB r	TGTGATTGTCTTTCTTCTGCCG	qRT-PCR
ATP6V1H f	GGAAGTGTCAGATGATCCCCA	qRT-PCR
ATP6V1H r	CCGTTTGCCTCGTGGATAAT	qRT-PCR
CTSB f	AGTGGAGAATGGCACACCCTA	qRT-PCR
CTSB r	AAGAAGCCATTGTCACCCCA	qRT-PCR
CTSF f	ACAGAGGAGGAGTTCCGCACTA	qRT-PCR
CTSF r	GCTTGCTTCATCTTGTTGCCA	qRT-PCR
GAPDH f	TGCACCACCAACTGCTTAGC	qRT-PCR
GAPDH r	GGCATGGACTGTGGTCATGAG	qRT-PCR
MonodoA f	TGA GAT GTG CTT CAT CTG CC	qRT-PCR
MonodoA r	CAGAGCCCCAGTCCTCAA	qRT-PCR
ChREBP f	GTCACGAAGCCACACACG	qRT-PCR
ChREBP r	GAGACAAGATCCGCCTGA AC	qRT-PCR
UVRAG f	TGGAGTCCCTAGTCCATGTTG	qRT-PCR
UVRAG r	AGGAGGGGAGAAGTTGCAGT	qRT-PCR
WIPI 149 f	GCACAATCTCCCCTGAAGTC	qRT-PCR
WIPI 149 r	CCTCCTGGATATTCCTGCAA	qRT-PCR
CLCN7 f	TGATCTCCACGTTCACCCTGA	qRT-PCR
CLCN7 r	TCTCCGAGTCAAACCTTCCGA	qRT-PCR
LAMP1 f	ACGTTACAGCGTCCAGCTCAT	qRT-PCR
LAMP1 r	TCTTTGGAGCTCGCATTGG	qRT-PCR
MCOLN1 f	TTGCTCTCTGCCAGCGGTACTA	qRT-PCR
MCOLN1 r	GCAGTCAGTAACCACCATCGGA	qRT-PCR
VPS18 f	TCATCCTTACGTCCCAGCTC	qRT-PCR
VPS18 r	AATCAGCTGTGCATGAGCCT	qRT-PCR
ATP6Voe1 f	CATTGTGATGAGCGTGTTCTGG	qRT-PCR
ATP6Voe1 r	AACTCCCCGGTTAGGACCCTTA	qRT-PCR

Supplementary Table 4: The Numbers of Differentially Expressed Genes in Different Comparisons.

**Comparison***	**Total no. of Differentially Expressed Genes (DEG)**	**No. of upregulated DEG***	**No. of downregulated DEG***
mml-1 vs N2	239	108	131
mxl-2 vs N2	1913	1174	739
hlh-30 vs N2	381	291	90
glp-1 vs N2	10806	4944	5862
glp-1;hlh-30 vs *glp-1*	543	206	337
glp-1 mml-1 vs *glp-1*	1360	670	692
glp-1 mxl-2 vs *glp-1*	1585	730	855
Intersection of *mml-1* vs N2 and *mxl-2* vs N2	165	67	90
Intersection of *glp-1mml-1* vs *glp-1* and *glp-1mxl-2* vs *glp-1*	827	445	382
Intersection of *glp-1;hlh-30* vs *glp-1*, *glp-1mml-1* vs *glp-1* and *glp-1mxl-2* vs *glp-1*	230	96	106

* In genotype A vs. genotype B, no. of upregulated or downregulated genes in genotype A compared to genotype B were counted.
